# Causally Investigating Cortical Dynamics and Signal Processing by Targeting Natural System Attractors With Precisely Timed (Electrical) Stimulation

**DOI:** 10.3389/fncom.2019.00007

**Published:** 2019-02-19

**Authors:** Dmitriy Lisitsyn, Udo A. Ernst

**Affiliations:** Computational Neuroscience Lab, Institute for Theoretical Physics, Department of Physics, University of Bremen, Bremen, Germany

**Keywords:** signal routing, selective attention, intracortical microstimulation, phase-response curves, communication-through-coherence, gamma-oscillations, visual cortex, model

## Abstract

Electrical stimulation is a promising tool for interacting with neuronal dynamics to identify neural mechanisms that underlie cognitive function. Since effects of a single short stimulation pulse typically vary greatly and depend on the current network state, many experimental paradigms have rather resorted to continuous or periodic stimulation in order to establish and maintain a desired effect. However, such an approach explicitly leads to forced and “unnatural” brain activity. Further, continuous stimulation can make it hard to parse the recorded activity and separate neural signal from stimulation artifacts. In this study we propose an alternate strategy: by monitoring a system in realtime, we use the existing preferred states or attractors of the network and apply short and precise pulses in order to switch between those states. When pushed into one of its attractors, one can use the natural tendency of the system to remain in such a state to prolong the effect of a stimulation pulse, opening a larger window of opportunity to observe the consequences on cognitive processing. To elaborate on this idea, we consider flexible information routing in the visual cortex as a prototypical example. When processing a stimulus, neural populations in the visual cortex have been found to engage in synchronized gamma activity. In this context, selective signal routing is achieved by changing the relative phase between oscillatory activity in sending and receiving populations (communication through coherence, CTC). In order to explore how perturbations interact with CTC, we investigate a network of interneuronal gamma (ING) oscillators composed of integrate-and-fire neurons exhibiting similar synchronization and signal routing phenomena. We develop a closed-loop stimulation paradigm based on the phase-response characteristics of the network and demonstrate its ability to establish desired synchronization states. By measuring information content throughout the model, we evaluate the effect of signal contamination caused by the stimulation in relation to the magnitude of the injected pulses and intrinsic noise in the system. Finally, we demonstrate that, up to a critical noise level, precisely timed perturbations can be used to artificially induce the effect of attention by selectively routing visual signals to higher cortical areas.

## 1. Introduction

With evolving technology, new and promising techniques to interfere with the brain natural activity have played a crucial role in moving from correlational to causal links between neuronal activity and behavior (Tehovnik et al., 2006; Logothetis et al., 2010; Fenno et al., 2011). Crucially, the same techniques are used clinically to treat pathological injuries and disorders (Martin et al., 2003; Benabid et al., 2009; Fisher et al., 2010; Berényi et al., 2012). The development of perturbation technology, among many others, includes ablations of cortical and subcortical targets, chemical lesions, reversible inactivations, transcranial direction current stimulation (tDCS), transcranial magnetic stimulation (TMS), intracortical microstimulation (ICMS), and finally the fairly recent and exciting optogenetic techniques (Wurtz, 2015). This advancement of tools has provided increasingly higher temporal and spatial perturbation precision, allowing for more intricate control over neural activity, which in turn has supported progressively stronger conclusions about the neuronal mechanisms underlying cognition.

While non-invasive techniques such as tDCS and TMS ease clinical applicability, the effects of their stimulation unfortunately lack spatial precision. Invasive techniques, in particular, ICMS and optogenetics allow for precise temporal and spatial resolution, providing the ability to deliver a single short and temporally precise perturbation at a precise location in the brain, which in turn, should greatly increase the ability to accurately affect and control neural circuits. However, the effect of such a single short perturbation can be very short-lived and, crucially, it can vary greatly in dependence on the state of the neural system at the pulse onset. Because of this, many perturbation paradigms have opted to either use a very strong pulse, essentially resetting and disrupting the activity of the target network, or to use a continuous or repetitive-pulse stimulation in order to establish and maintain a desired effect. For instance, a series of seminal studies (Cardin et al., 2009; Siegle et al., 2014) entrained a local population in the barrel cortex of mice with a rhythmic optogenetic train of pulses at 40 Hz. By delivering a vibrissa stimulation at different phases of the entrained population cyclic activity, the researchers showed that the neural population response as well as the rodent behavioral performance depends on the phase at which the whisker stimulation stimulus arrives to the population. In a more recent study, Ni et al. (2016) used a similar technique to show how an optogenetically induced neural rhythm modulates the gain of spike responses and behavioral reaction times in response to visual stimuli in cats.

Using continuous stimulation serves its role as a powerful research tool, however it also brings up a number of concerns. First, in some cases, stimulation can effectively destroy and suppress any ongoing local processing (Logothetis et al., 2010). Even if it does not lead to full suppression, in addition to achieving a desired effect, continuous stimulation may interfere and contaminate the relevant neural signals. Further, in many cases, when analyzing the activity recorded during the stimulation, it becomes hard, if not unfeasible, to separate the stimulation artifacts from the relevant neural data. Finally, such an approach explicitly forces the neural system to remain in some desired network state, resulting in artificial dynamics and making it questionable what we learn about processing during natural activity.

In this study, we propose to use an alternate strategy. Rather than using continuous stimulation in order to sustain a desired state of the neural network, we wish to utilize a single precise pulse in order to push the system into one of its (potentially) existing preferred states (Tsodyks et al., 1999). If the network is pushed into one of its attractors, the natural tendency of the system to remain in such a state extends the duration of the effect of the pulse, which opens up a larger window of opportunity to observe the consequences on cognitive processing. Crucially, it becomes necessary to monitor the system in real time in order to be aware of the system state and to deliver just the right stimulation at just the right time, resulting in a closed-loop paradigm.

The brain's rhythmic activity and synchronization phenomena provide a perfect test-bed for our approach. Brain rhythms have been at the center of neuroscience research since they were first observed with the invention of EEG (electroencephalography) over a century ago (Coenen et al., 2014). Neural oscillations are generated at specific frequencies, coexisting with background noise (non-oscillatory) activity. They can be observed at multiple scales, from the activity of a single neuron to the coordinated output of large neuronal networks (Varela et al., 2001). Further, distinct neural populations can entrain each other, exhibiting coupled states and synchronized activity (Singer, 1999; Varela et al., 2001). Research has shown that such neural synchrony plays a crucial role that underlies many cognitive processes, such as perceptual grouping (Schmidt et al., 1997), working memory (Sarnthein et al., 1998), and information routing of signals throughout the brain (Fries, 2005). Clinically, abnormal levels of synchrony have been linked to pathological disorders, such as schizophrenia, autism, Alzheimer's, and Parkinson's (Uhlhaas and Singer, 2006).

Approaching the brain's rhythmic activity and synchronization phenomena from a perspective of non-linear dynamics provides useful inferences on neural oscillator activity (Guevara Erra et al., 2017). First, oscillatory synchronization collapses the normally high dimensional dynamics of neural dynamics into a low dimensional set of attractor states. Further, if a neural system can be modeled using self-sustained, oscillators, a perturbation inserted at a specific phase of a cycle would evoke a consistent phase-shift in the oscillator's activity—an effect that is captured by a phase-response-curve (PRC) (Schultheiss et al., 2011; Canavier, 2015). Numerous experimental studies have found evidence for PRCs *in vitro* (Akam et al., 2012) and as well as *in vivo* (Velazquez et al., 2007, 2015; Voloh and Womelsdorf, 2016).

In the present study, through a modeling approach, we develop a method to explore the feasibility of utilizing PRCs in order to shift the synchronization of a system into a desired state. First, we choose to model selective information routing in the visual cortex, between V1 and V4 cortical areas. A prominent mechanism explaining how information routing occurs, communication through coherence (CTC), relies on the inherent oscillatory dynamics of neural activity and postulates that neural populations establish favorable and unfavorable information routing states through frequency-specific phase-locking (Fries, 2005, 2015). In support of this hypothesis, experimental studies have shown strong evidence for gamma-band synchronization between sending V1 and receiving V4 neural populations during a visual attention task (Bosman et al., 2012; Grothe et al., 2012). Once a favorable synchronization state is established, rhythmic bursts of V1 spikes arrive to V4 during its excitability peaks, increasing the likelihood that further spikes are evoked leading to effective signal routing. On the other hand, if the V1 and V4 populations establish an unfavorable phase state relationship, the V1 spikes arrive to V4 during the excitability troughs and hence should fail or at least be less effective in evoking further activity.

We begin with a model of an isolated neural oscillator and then expand to a more realistic system of multiple coupled populations, constructed to exhibit the synchronization and information routing phenomena observed in the visual cortex. We explicitly measure the information content in the model to evaluate the effect of signal contamination caused by the stimulation in relation to the magnitude of the injected pulses and intrinsic noise level of the system. Further, we vary the background noise level to investigate how increased stochasticity affects the phase-response properties the system and hence our ability to control it. We demonstrate that up to a critical noise level, precisely timed perturbations can be used to “simulate” the effect of attention by selectively routing a visual signal to higher cortical areas and identify optimal pulse strengths required to achieve this goal.

## 2. Results

In the first part of this section, we present the model of a single cortical column as the basic building block of our framework. Further, we introduce the techniques needed to monitor oscillatory dynamics, and demonstrate how to use them to control single oscillators to maintain a desired system state. Taken together, these considerations pave the way for interacting with a more realistic, hierarchical cortical network in Part II of this section.

### 2.1. Part I: Stimulating a Cortical Column—Basic Concepts

#### 2.1.1. Cortical Column Model

##### 2.1.1.1. Model structure and dynamics

For representing one cortical column, we construct a recurrent network with 800 excitatory and 200 inhibitory, conduction-based quadratic integrate-and-fire neurons. Their membrane potentials *V* evolve according to the differential equation

(1) $$C_{m}\dot{V}=p_{2}V^{2}+p_{1}V+p_{0}+g_{e}(V-V_{e})+g_{i}(V-V_{i})+\sigma_{n}\eta (t).$$

Here, *C*_*m*_ is the membrane capacitance, *V*_*e*_ and *V*_*i*_ are the reversal potentials and *g*_*e*_ and *g*_*i*_ the corresponding conductances for excitatory and inhibitory input currents, and η(*t*) is 1/f (pink) noise with magnitude σ_*n*_. If the membrane potential *V* crosses the threshold *V*_*thresh*_, a spike is generated and delivered to all connected neurons, and *V* is reset to *V*_*rest*_.

**Figure 1:**
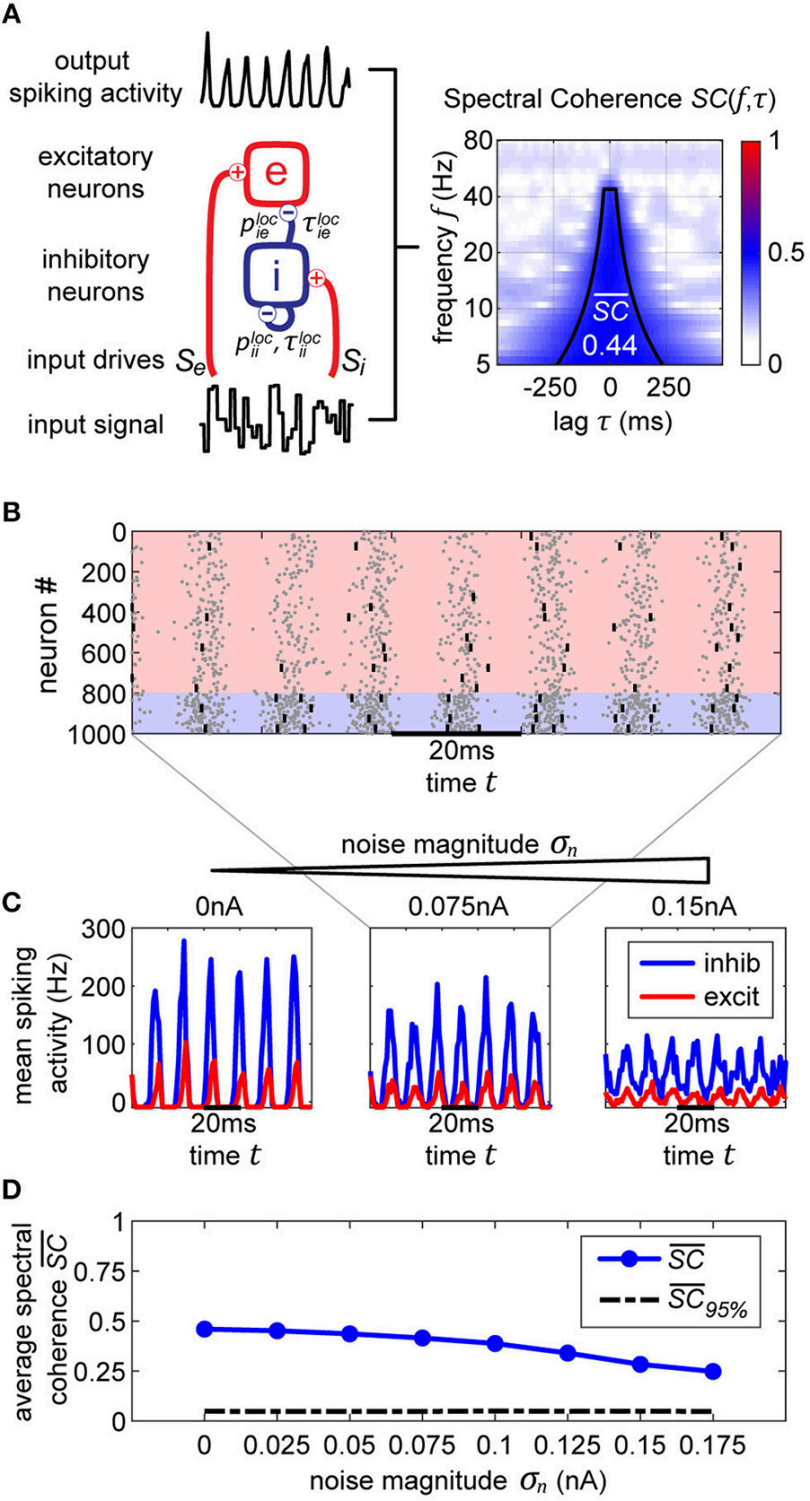
Single oscillator model and activity. **(A)** A single cortical column is represented by a neural oscillator, consisting of an excitatory and an inhibitory neuron population. It is driven by an input signal containing a time-varying amplitude modulation. The inhibitory population projects onto itself and onto the excitatory neurons, resulting in an ING mechanism which produces cyclic population activity in the gamma frequency range (60–75 Hz). To evaluate the signal routing ability of the network, we assess the spectral coherence SC(*f*, τ) between the input signal modulation and the excitatory output activity for different frequencies *f* and signal time lags τ. An cumulative input signal information measure $\bar{SC}$ (white text) is computed by pooling across the relevant time-frequency range within the cone of interest (solid black lines). **(B)** A raster plot of the all spiking activity withing the system for the medium amount of background noise σ_*n*_ = 0.075 nA. Neurons 1–800 are excitatory (red shading) and 801–1,000 are inhibitory. One in fifty neurons is marked by a small black bar, in order to highlight the spiking activity of a few individual neurons. **(C)** Samples of excitatory and inhibitory population activity for increasing internal noise levels are displayed for multiple background noise conditions. At zero noise, oscillatory spiking activity is very regular—large population bursts are followed by periods of silence. With increasing noise, activity gets more irregular and less phase specific. **(D)** We show how input signal modulation contribution to the neural activity $\bar{SC}$ decreases with increasing internal noise σ_*n*_. The dashed line at the bottom indicates the 95% chance level, calculated by pairing up the network activity with surrogate input signals.

Synaptic term equations and all the relevant parameter values are presented in Table 1. Connections exist from the inhibitory population to itself, with projection probability $p_{ii}^{loc}=0.5$ and corresponding delay $\tau_{ii}^{loc}=5$ ms, and from the inhibitory to the excitatory populations, with projection probability $p_{ie}^{loc}=0.5$ and corresponding delay $\tau_{ie}^{loc}=5$ ms (Figure 1A). The high link probability of the inhibitory neurons reflects the dense connectivity of the inhibitory interneurons found in the cortex (Packer and Yuste, 2011). The neuron and coupling parameters are set to emulate realistic neurons, in accordance with Bartos et al. (2002) (for details on the implementation and parameters see Methods section). In our case, having 5 ms delays allows the network to generate gamma frequency oscillations (Figures 1B,C) by means of an ING-mechanism (Tiesinga and Sejnowski, 2009).

**Table 1:** Neuron and synaptic connection parameters.

**Variable** value	*A*_*e*_ 2.88 × 10^−4^cm^2^	*A*_*i*_ 1.2 × 10^−4^cm^2^	*p*_0_ 3.89 × 10^−9^ A	*p*_1_ 1.30 × 10^−7^ A/V
**Variable** value	*p*_2_ 1.08 × 10^−6^ A/V^2^	*V*_*e*_ 0 mV	*V*_*i*_ −75 mV	*V*_*thresh*_ −56.23 mV
**Variable** value	*V*_*reset*_ −67 mV	τ_*e*_ 3 ms	$\tau_{i}^{1}$ 1.2 ms	$\tau_{i}^{2}$ 8 ms
**Variable** value	χ_1_ 0.9	χ_2_ 0.1	ω_*e*_ 0.4 nA	ω_*i*_ 1.2 nA

*The parameters are taken to emulate biophysically-realistic neurons, in accordance with Bartos et al. (2002). These parameters were specifically derived from neural data, corresponding to neurons responsible for generative gamma oscillations in the hippocampus. p_0, 1, 2_ were found by a mathematical reduction of the Hodgkin-Huxley model (Abbott and Kepler, 1990)*.

Both populations are driven by afferent connections delivering excitatory input with time-varying rates *S*_*e*_(*t*) and *S*_*i*_(*t*), realized by inhomogeneous Poisson processes. We scaled the mean rate and driving magnitude of the afferent input such that we achieve a relatively high firing rate of 60 Hz for the inhibitory units, and a significantly lower rate of 15 Hz for the excitatory units reflecting the typical differences found between the firing rates of the neuron types in the cortex (Vinck et al., 2013).

##### 2.1.1.2. Quantifying stimulus representation

When interacting with a cortical network by external electric stimulation, we pursue two goals: Assessing the implied changes in dynamical network states, and quantifying the impact on function, i.e., the representation and processing of visual information. For the latter goal, we adopt a method which was used successfully to quantify selective signal transmission (“gating”) in dependence on the attentional state (Harnack et al., 2015; Grothe et al., 2018). The main idea of this method is to modulate the visual (input) signal by a random change in its amplitude (“flicker”), and to compare the output of a neural population with the input flicker signal by computing a frequency-resolved correlation using spectral coherence (SC). In our case, we modulated the external drive with mean rate S${}_{x}^{0}$, *x*∈{*e, i*} by a flicker signal *F*_*x*_ via

(2) $$S_{x}(t)=S_{x}^{0}(1+\sigma_{F}F_{x}(t)).$$

*F*_*x*_ was sampled from a uniform distribution between [−1, 1], changing every 10 ms, corresponding to the experimental flicker signals used in Grothe et al. (2018) where a luminance of a stimulus changed every frame at 100 frames per second. The strength of flicker modulation was set to σ_*F*_ = 0.10. This modulation is passed onto the spiking rates of the driven neural populations (Figure 1A). Note that even though the background 1/f noise η(*t*) and the flicker modulation *F*_*x*_(*t*) appear to have similar effects on the model, the flicker changes at a much lower rate and its magnitude is kept consistent throughout all the simulations, whereas the magnitude of background noise η(*t*) is used to change the noise level of the system. By design, the flicker is the signal we track throughout the network, and η(*t*) constitutes intrinsic, interfering noise that affects the cleanliness of oscillations and overall stability of network states.

To assess the input flicker modulation contribution to the neural activity, we utilize spectral coherence (SC). This method allows to investigate the linear contribution of the input to network's activity and was successfully used experimentally to study similar selective processing in Grothe et al. (2018). In our study, it provides a simple proxy to evaluate how well we can control the system and the level of signal degradation due to perturbations. This does not exclude that stimuli information is also encoded in other ways such as population or temporal coding, but suffices to compare the effects of “simulated” attention by ICMS to “physiological” attention.

First, we compute the spectrograms of the input signal and the spike output using a wavelet transform with Morlet kernels. The transform yields complex valued coefficients *W*_*z*_(*f, t*) representing the amplitude and phase of a signal *z*(*t*) around the frequency band *f* at time *t*. By evaluating the normalized cross-correlation between the spectrograms of *x*(*t*) and *y*(*t*) we obtain the spectral coherence measure *C*_*xy*_(*f*, τ), where *f* is the frequency and τ is the delay between the two signals:

(3) $$C_{xy}(f,\tau )=\frac{\sum_{i}W_{x}^{*}(f,t_{i})\;\cdot \;W_{y}(f,t_{i}+\tau )}{\sum_{i}|W_{x}(f,t_{i})|\;\cdot \;\;\sum_{i}\left|W_{y}(f,t_{i}+\tau )\right|},$$

Due to the normalization terms in the denominator, the values of *C*_*xy*_ lie between zero and one.

If neurons are driven well by the external stimulus, experimental data (Grothe et al., 2018) and model simulations (Figure 1A) reveal that the input signal can be tracked in the population activity of a cortical column in V1 (or V4) up to frequencies of about 45 Hz (or 25 Hz). Hence, in order to obtain a cumulative measure of input signal contribution to the neural activity, we defined a cone-of-interest whose upper frequency limit was selected to be at 45 Hz, and whose temporal range was defined as ±7/6*f* around τ = τ_*xy*_, where τ_*xy*_ is the delay between input signal *x* and neural output *y*. We pooled across the relevant frequencies *f* and time lags τ within the cone of interest, to compute a single spectral coherence score ${\bar{SC}}_{xy}$.

##### 2.1.1.3. Gamma oscillations and noise

In a typical experimental situation, it is impossible to assess the output signal of a specific neural population directly. Instead, the measurement is confounded by both, measurement noise and noise induced by background activity or by contributions from neighboring circuits. For interacting with the brain, it is therefore essential to quantify the impact of noise on the assessment of the current system state and to determine limits up to which successful control is still possible. We therefore introduced internal noise via the additional term σ_*n*_η(*t*) in Equation (1) (Fourcaud and Brunel, 2002). η(*t*) is 1/*f* noise with standard deviation equal to 1, making σ_*n*_ represent the magnitude of the noise. Every single neuron unit receives its own unique noise input. By changing the magnitude σ_*n*_, we control the overall level of noise in the entire system.

In Figure 1C in the top three plots, we display model activity at different noise levels. With zero noise level we clearly see oscillations within the Gamma frequency range, with low jitter and high regularity and phase specificity—inhibitory and excitatory populations of neurons both evoke concentrated bursts of spikes followed by periods of silence. Increasing the noise renders oscillations more irregular and less phase-specific, and decreases peak amplitudes. Also, oscillation frequency increases from 60 Hz for the zero noise condition to 75 Hz for 0.15 nA. In order to maintain a stable cyclic activity with a constant frequency, the ratio of inhibitory and excitatory post-synaptic currents needs to stay consistent within each population of neurons (Buzsáki and Wang, 2012). Increasing the magnitude of noise inherently raises the firing rate of neurons. Since our units are recurrently coupled, a change in average firing rate upsets the inhibition-excitation ratio of the system, which results in a dramatic change in activity. To counteract this effect, for each noise level, we update the magnitude of driving rates *S*_*e*_ and *S*_*i*_ to provide just the right amount of input drive to excitatory and inhibitory units to maintain firing rates consistent with physiological evidence, i.e., an average of 15 Hz for excitatory and 60 Hz for inhibitory units (see Methods section).

For noise levels of about 0.1 nA, we observe signals similar to physiological findings (Grothe et al., 2018). To cover a realistic range, we investigated noise levels from σ_*n*_ = 0 nA up to σ_*n*_ = 0.175 nA. Crucially, as can be expected, the input signal representation as quantified by $\bar{SC}$ becomes worse with increasing noise, although it stays well above the significance level (Figure 1D).

#### 2.1.2. Tracking Oscillations and Stimulation Effects

##### 2.1.2.1. Real-time phase tracking

For a targeted interaction with a neural system, we have to assess its internal state in real-time. In our case, the internal state is characterized by the current phase of an ongoing oscillation (in the Gamma frequency range). Consequently, we will have to determine this phase as precisely and timely as possible.

**Figure 2:**
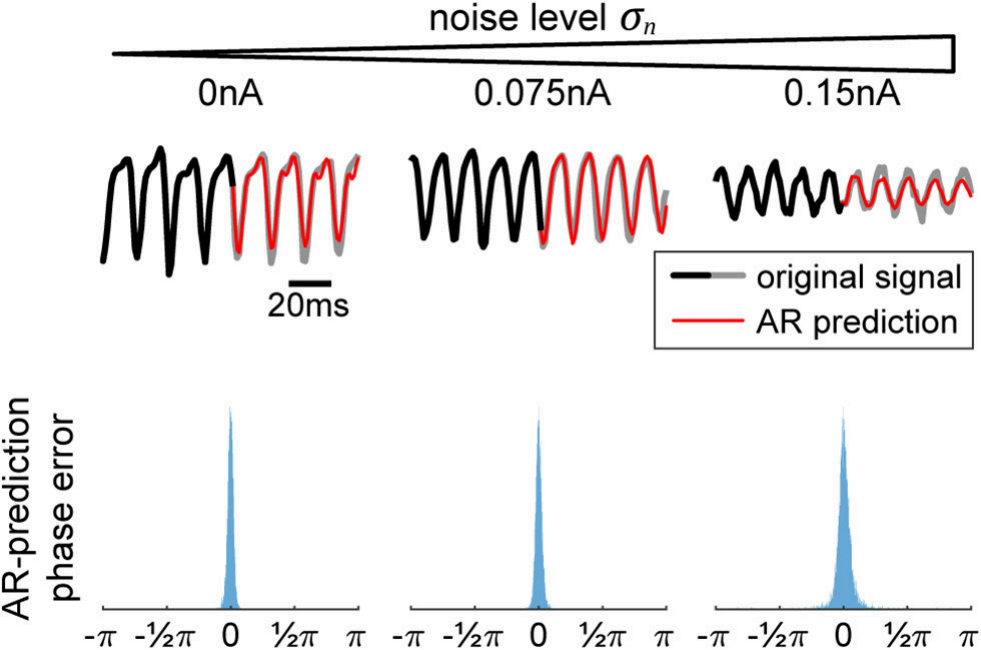
Autoregression (AR) signal prediction and phase error. In order to extract real-time phase or the phase of a signal prior to a perturbation, we utilize AR in order to forecast signals into the future before using typical offline methods (Hilbert transform). In the **(top)** row, we show a few current signals generated by our model for increasing levels of noise. The black-to-gray line shows the original signal and the red line shows the AR prediction. In the **(bottom)** row, we show the corresponding distributions of differences between offline vs. real-time phase, showing the efficacy of the method.

Tracking the phase of a signal in real-time imposes the constraint that only data from the past can be used for phase measurement, whereas the typical offline phase measurement algorithms rely on utilizing past and future data for an accurate estimate of the instantaneous phase at that time point. Hence, we utilize use a phase-extraction scheme motivated by Chen et al. (2013) that relies on using autoregression (AR) in order to forecast the signal forwards (Figure 2, top row). The AR model has been found to perform well in forecasting noisy signals with power spectrum limited to certain frequencies (Blinowska and Malinowski, 1991), making it adequate for our data.

In order to use the AR model, it must first be trained on data without any perturbations. Once the model is acquired, it is used to extend the signal into the future, allowing us to use any of the typical offline methods for phase extraction. In our case, we utilize the Hilbert transform. We apply a zero-phase bandpass filter with bandstops at the halfway points found in the power spectrum to obtain the gamma component of the signal without distorting its phase. Then, the data is passed through a Hilbert transform (Boashash, 1992), providing us with the complex analytical signal. The argument of the analytical signal reveals the instantaneous gamma phase. The narrow range of the bandpass filter is necessary, since the instantaneous phase only becomes accurate and meaningful if the filter bandwidth is sufficiently narrow (Nho and Loughlin, 1999). The difference between the realtime and offline phase extraction shows to be sufficiently small, demonstrating the efficacy of the method (Figure 2, bottom row), and obliviating the need to revert to more elaborate phase estimation schemes such as using multiple band-pass filters with slightly different filter parameters (Mortezapouraghdam et al., 2018).

In addition to allowing us to extract realtime phase, the same method is also applied to neural signals just prior to an input pulse to determine the phase of the ongoing oscillation before it is affected by the systems response to the perturbation. A similar method relying on AR was utilized specifically for this reason in Ni et al. (2016).

##### 2.1.2.2. Phase-response curves

**Figure 3:**
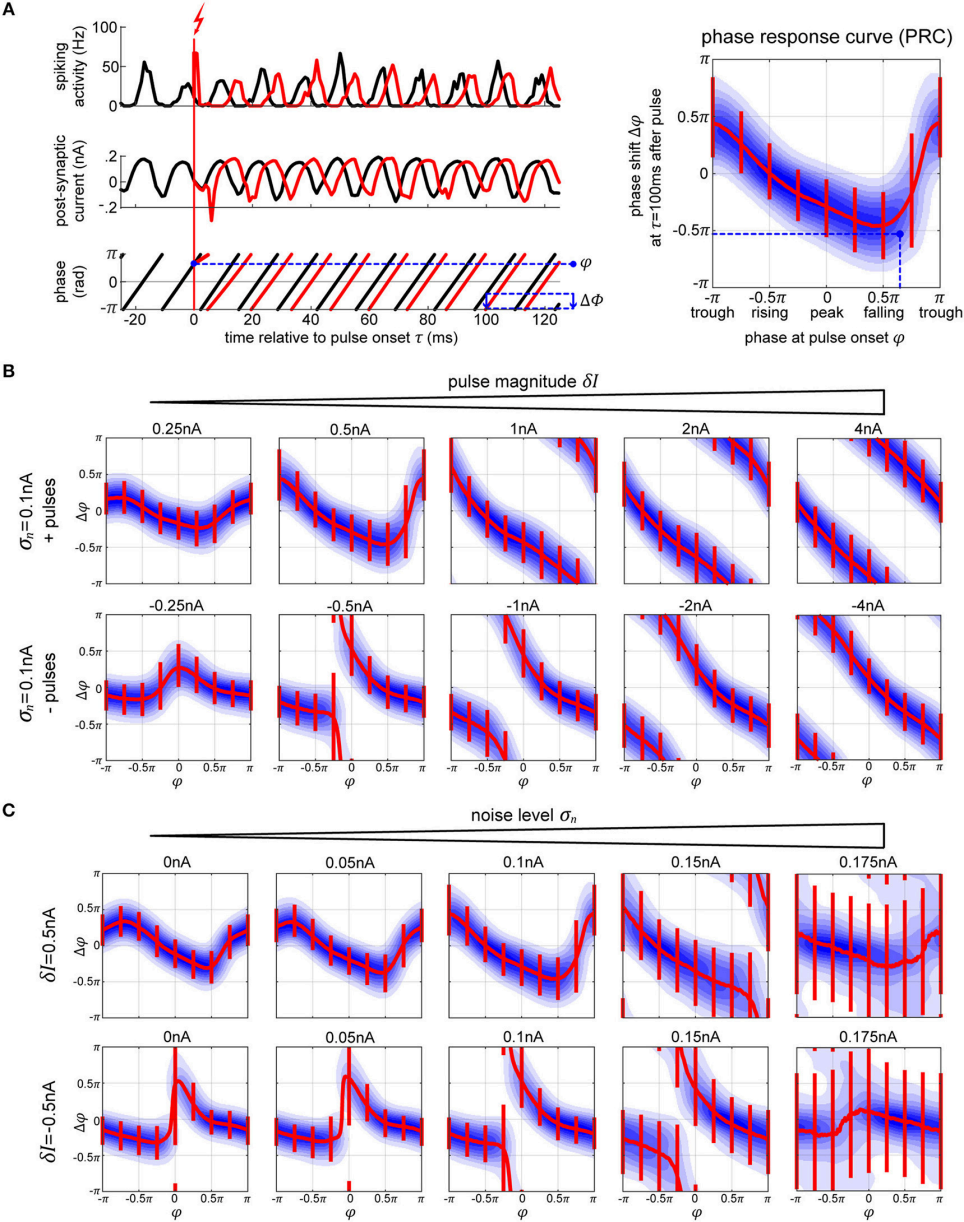
Phase response curves. **(A)** The plots on the left (top and middle row) show the activity of the excitatory population (in black) and how a single perturbation applied to the oscillator changes both activity and post-synaptic current (in red). From the corresponding phase dynamics (bottom row), we capture the phase φ at the pulse onset, and the resulting phase shift δφ 100 ms later (blue arrow). This gives us a single data point (marked in black) in the PRC space on the right. By repeating this procedure we obtain the distribution of pulse responses indicating the PRC and its variability due to internal and external noise sources. The blue shading in the plot corresponds to the probability density of many runs, with darker blue corresponding to higher probability. **(B,C)** We show multiple PRCs across different conditions—varying pulse magnitude **(B)** and internal noise level **(C)**. In each plot, the thick red line represents circular mean of the phase shift across the pulse onsets, while the thin red lines indicate the corresponding 25th and 75th percentiles. At low pulse strengths, the resulting PRC shows a smooth biphasic relationship—pulsing at the peak [0 < φ(*t*_*onset*_) < 0.5π] results in a negative phase shift (delay) and pulsing at the trough [−π < φ(*t*_*onset*_) < −0.5π] gives a positive phase shift. As we increase the strength of the perturbation the magnitude of the phase shift increases. At sufficiently high pulse magnitude of either polarity, a perturbation leads to a complete phase reset.

Using stimulation pulses, our goal is “push” a neural system toward particular states and quantify the impact of such a “configuration change” on information processing. For an oscillatory system, when a perturbation occurs at a specific phase of its cyclic activity, the following oscillatory activity is shifted by a consistent amount. This can be quantified by a phase-response-curve (PRC) (Smeal et al., 2010; Schultheiss et al., 2011; Canavier, 2015) by tabulating the phase shift Δφ induced by a perturbation in dependence on the phase φ at pulse onset (Figure 3A). Conversely, a PRC can be used to determine the “right” time for a stimulation in order to shift the system's phase by a desired amount.

We simulate electric stimulation by injecting a square pulse of current of 1 ms duration into all the neurons within the oscillator. The pulse was intended to emulate intracortical microstimulation (ICMS), affecting the population of local neurons indiscriminately. We tested depolarizing (positive pulses, exciting the neurons) and hyperpolarizing (negative pulses, inhibiting the neurons) pulse polarities at multiple pulse strengths δ*I*, from 0.25 to 4 nA. To collect the PRC curve data, first, we run the model for a total time *T* without any stimulation pulses (Figure 3A, left panel, black curves). Using real-time phase measurement, this data allows us to tabulate normal, unpulsed phase progression φ(*t*). For assessing the impact of perturbation on phase, we again run the model for time *T*, now pulsing at random points in time, and obtain the pulsed phase progression φ_δ*I*_(*t*) (Figure 3A, left panel, red curves). The resulting phase shift observed after a delay time τ is then given by Δφ(τ) = φ_δ*I*_(*t*_*onset*_ + τ) − φ(*t*_*onset*_ + τ). Except for very simple or idealized systems, Δφ is typically not independent of τ. In particular, one distinguishes between the *immediate* PRC for τ = 0^+^, and the *permanent* PRC for large τ (Prinz et al., 2003).

Since we consider a stochastic dynamical system, one can not directly obtain a PRC from network simulations. Instead we repeated the described procedure for sufficiently many *t*_*onset*_'s to first obtain a phase-response probability density function ρ_τ_(Δφ|φ) (Figure 3A, right panel, blue shading). By taking the circular mean across Δφ, one can condense ρ_τ_ into a mean PRC ${\bar{\Delta \varphi }}_{\tau }\left(\varphi \right)$ (Figure 3A, right panel, red line), whose inverse gives the appropriate onset phase(s) φ which achieve(s) on average a phase shift Δφ at time τ after giving the pulse.

Note that this inverse mapping does not have to be unique, nor does it have to exist for any desired phase shift, especially for low pulse strengths. In theory, one can realize any desired phase shift by using a sequence of (small) shifts into the right direction, but since we have to cope with a noisy dynamics inducing frequency jitter, we typically aim at achieving a desired shift with as few pulses as possible.

Note that the phase shift Δφ does not occur immediately. Rather, following the stimulation, the network takes time to stabilize and settle back into its normal cyclic activity, similar to what has been described as “permanent resetting” in the case of PRCs for individual neurons (Prinz et al., 2003). In the single oscillator model, it takes around 2–3 cycles (around τ = 30 ms) for the network to settle into its new stable phase state. Following this time point, the mean phase shift stays consistent, however, the variability goes up, due to the activities' intrinsic fluctuations in frequency.

For weak perturbations (δ*I* = 0.25−1.0 nA pulse magnitude), the resulting phase offsets are small, resulting in a smooth biphasic PRC. The negative and positive pulses cause shifts into opposite directions (Figure 3B, compare top and bottom rows of first plots on the left). However, as the strength of the pulse increases, the phase-shifts increase as well, until they look the same and a complete phase reset occurs resulting in a PRC that approaches the shape of a straight line (Figure 3B, plots on the right). With a strong negative/hyperpolarizing pulse, both the excitatory and inhibitory neurons are reset to their steady state and the whole system is silenced. When a strong positive/depolarizing pulse is delivered, both populations of the network discharge a large volley of spikes, which is then followed by a strong hyperpolarization τ_*ii*_ = τ_*ie*_ = 5 ms later due to the connections from the inhibitory neurons, which essentially acts as a strong negative pulse onto the system. In each case, the neurons are reset to the steady state, taking the network a predictable amount of time to recover back to its oscillatory activity, regardless of onset phase of the perturbation.

As we increase the internal noise of the model, the variability of the PRC goes up with it. At a sufficiently high noise level, σ_*n*_≥0.175 nA, we no longer achieve stable or predictable phase shifts (Figure 3C), which means that the oscillator has become too unstable to exhibit phase-response properties (Guevara Erra et al., 2017). Additionally, when applying a specific magnitude of a pulse, its effect seems to increase (getting closer to a full phase-reset) with noise as well. In part, this is due to the fact that the amplitude of the oscillations in the noisy conditions are lower, meaning that the relative magnitude of the pulse to the oscillations gets higher with increasing noise.

#### 2.1.3. Controlling Oscillations

##### 2.1.3.1. Phase control procedure

**Figure 4:**
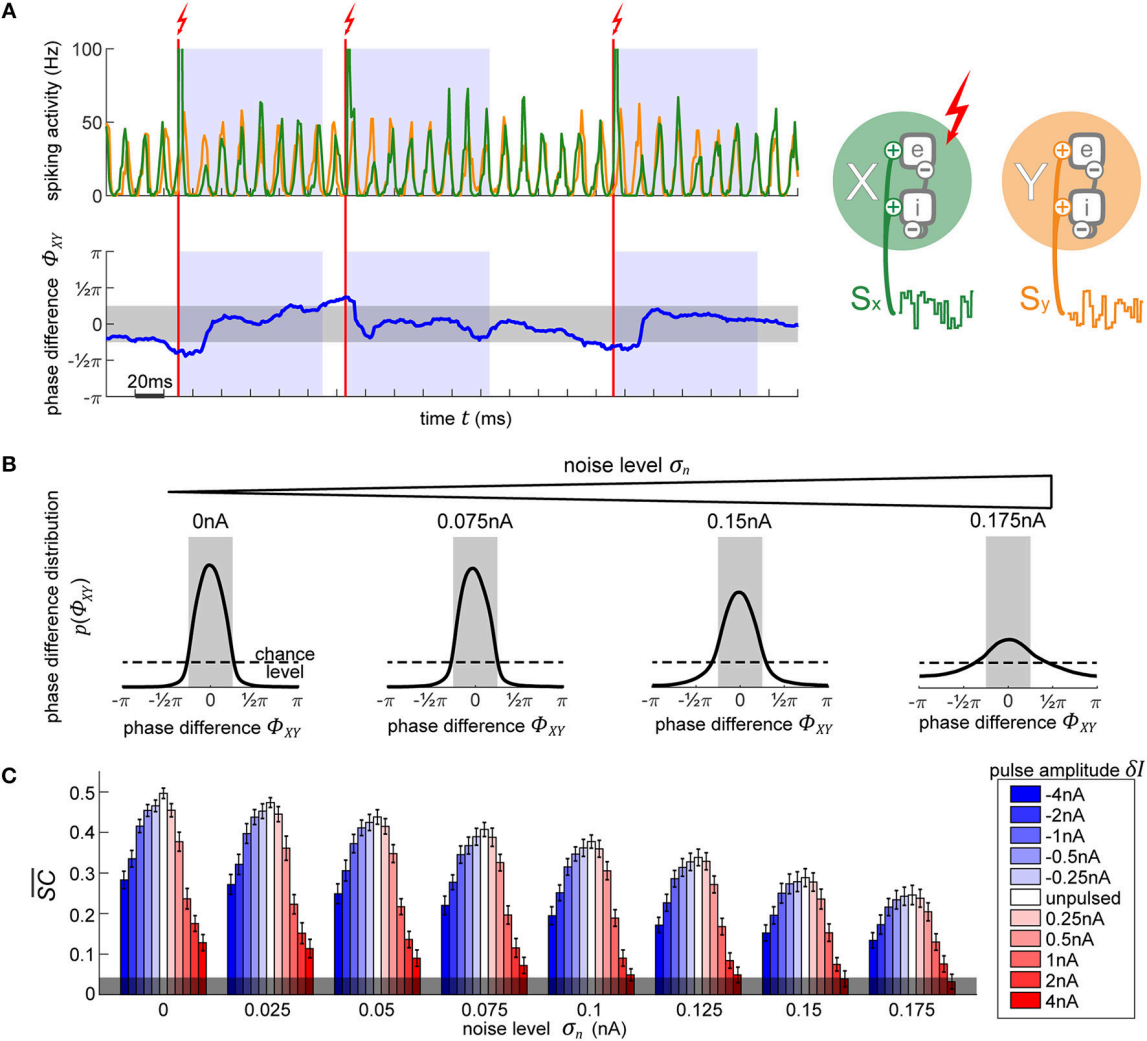
Using PRCs to synchronize two independent oscillators. **(A)** The top plot shows the diagram of the two oscillators next to their output activity traces, X (green) and Y (orange). As soon as the phase difference between the two oscillators (lower plot, blue line) exceeds 0.25π in either direction (region shaded in gray), the appropriate phase for pulse onset onto X in order to achieve the required shift to bring it back into synchronization with Y is determined. Once X is at this right phase, a pulse is applied (vertical red line). Following the stimulation pulse, a refractory period is induced for 100 ms during which no pulses are allowed (blue-shaded regions). **(B)** Evolution of X-Y phase difference distribution with increasing noise. With higher noise, the amount of time that the model spends in the desired state diminishes, as visible by the broadening distribution. The magnitude of the pulse does not affect the distributions, since any of the pulses are equally capable of causing the appropriate shift to maintain an already established and desired phase relationship between the oscillators. **(C)** Signal content $\bar{SC}$ for different pulse strengths (blue-red scale) across different background noise conditions. Stronger pulses cause larger and longer-lasting artifacts in activity which greatly reduce the signal information content measure $\bar{SC}$. Negative pulses consistently lead to less signal degradation than positive pulses of the same magnitude. The errorbars correspond to the SE of 10-s simulation runs. The gray shading at the bottom indicated the 95% chance level.

To test the ability to use the phase-response characteristics of our model, we employ the following task: we run two independent oscillators, X and Y, simultaneously. If we let them run without interfering, the phase difference between their activities Φ_XY_ = φ_X_−φ_Y_ performs a random walk, as their frequencies fluctuate independently from each other^1^. We want to pulse X to keep it synchronized with Y. To achieve this, first, we track their phases φ_X_ and φ_Y_ in realtime, using the AR model to forecast signal at each time point (see Figure 4A). Once Φ_XY_ surpasses the allowed threshold level (more than an eighth of a cycle difference, |Φ_XY_| > π/4), we apply a stimulation pulse at just the right phase in order to enact a shift in X's phase Δφ_X_ that is as close as possible to the required correction −ΔΦ_XY_. As soon as φ_X_ matches the desired onset phase, the stimulation current is given. After the pulse, we enforce a refractory period of τ_ref_ = 100 ms when no pulses are allowed in order to let the network settle and maintain its new phase relationship.

##### 2.1.3.2. Synchronizing two independent oscillators

In Figure 4B we show the resulting phase difference between X and Y, with the desired state shaded in gray. In the unpulsed control condition, due the inherent variability in the oscillator's frequency, their phase difference constitutes a random walk, resulting in a uniform distribution. Once the closed-loop procedure is applied, the difference of phases between the oscillators shows the desired distribution centered around the target phase state (-π/4 to +π/4). Notably, the strength of the pulse does not affect the distribution (hence, just one distribution shown for all pulse strengths in Figure 4B). Once the first few pulses bring the oscillators into the desired phase relationship, only small phase shifts are required in order to maintain the phase difference. Since any of the utilized pulse magnitudes are capable of achieving the required shifts, the final phase difference distribution is unaffected.

On the other hand, the model's inherent noise level plays a major role. As the noise increases, the ability of the pulsing procedure to maintain the desired state decreases. At the highest noise level, even though their PRC curve shows no reliable shifts, we still achieve a distribution centered around the desired phase difference. Note, that this is not due to any phase-response properties but is merely the effect of applying pulses to the network whenever it is not in the desired state, thus pushing it away from the “forbidden” state, and then letting it run passively whenever the desired state is achieved—the phase onset of the pulse does not matter.

Next, we use spectral coherence to assess the amount of input signal information that is present in the networks' output activity (Figure 4C). By pulsing the population, we degrade the signal content. With perturbations of higher magnitude, the amount of degradation increases appropriately. Notably, negative perturbation pulses (in blue) result in significantly less information degradation than the positive pulses (in red). The excitatory pulses evoke large bursts of spiking activity, which strongly diminishes the stimulus content measure, whereas the inhibitory pulses, at most, suppress the spiking activity to zero which results in less stimulus interference. At high noise levels and at a sufficiently high pulse magnitude (4 nA), the amount of signal information is no longer significant and falls below the 95% chance level at the bottom of the plot. Thus, if we want to use electrical stimulation for assessing information processing in the brain, we have to take care to use an appropriate pulse strength to not completely overpower the signals whose representations we desire to enhance.

### 2.2. Part II: Bistable Columnar Network

Here the techniques developed in the first part of our study will be applied to an established, prototypical columnar network implementing selective signal routing under attention. After briefly describing the model itself and its dynamics, we will first quantify how the model reacts to perturbation pulses applied to different parts of the system. Using this knowledge, we can finally interact with the model “cortex” in a meaningful way, simulating the effects of “natural,” physiological attention by using “artificial” pulses to selectively route external signals to neural target populations. Conversely, our results provide predictions which can be used in physiological experiments to specifically test the particular model setup and, on a more general level, hypotheses about the still debated neural mechanisms realizing communication-through-coherence.

#### 2.2.1. Structure and Dynamics of Columnar Network

##### 2.2.1.1. Setup and connectivity

We use the cortical column setup from Part I to construct a model composed of several interconnected oscillator modules, representing interactions between cortical columns in areas V1 and V4, similar to the work of Harnack et al. (2015). All the projections between the cortical columns originate from their respective excitatory subpopulation, reflecting the finding that inhibitory neurons have been found to form primarily local connections, whereas the excitatory neurons project to up- and down-stream visual areas (Stepanyants et al., 2009), and laterally to neighboring columns (Stettler et al., 2002).

**Figure 5:**
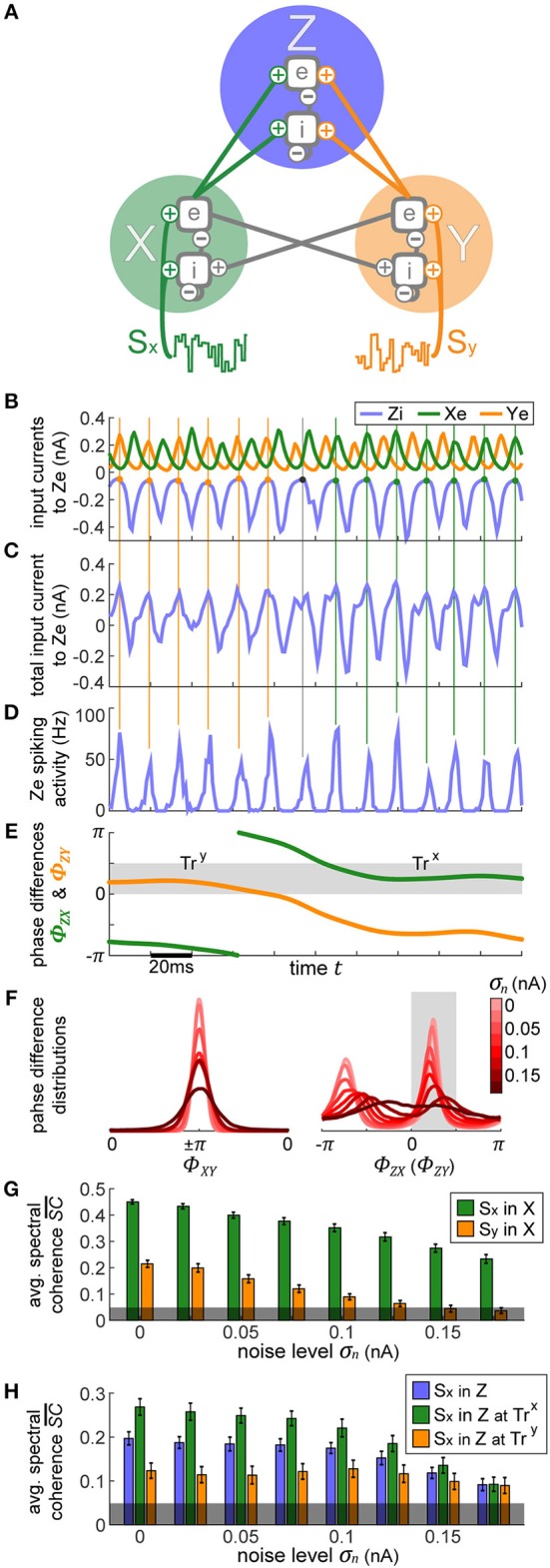
Bistable XYZ model. **(A)** The model consists of three cortical populations X, Y, and Z. X and Y form the lower layer of the model, each population is driven by its own input signal S_X_ and S_Y_. X and Y are connected laterally, set up such that the two populations' activities oscillate in antiphase. The outputs of X and Y drive the activity of Z which forms the upper layer. **(B–E)** In row **(B)**, we display the postsynaptic currents in Z_*e*_ as a result of the inputs it receives from X_*e*_, Y_*e*_, and Z_*i*_. X and Y's activity is consistently in antiphase. In row **(C)**, we show the sum of all these currents, which then leads to the spiking activity displayed in row **(D)**. Points of minimal inhibition from Z_*i*_ (i.e., peaks in the inhibitory Z_*i*_ current) are marked with vertical lines extending across the plots in order to track their location. Toward the beginning, these peaks align with the peaks of X_*e*_'s current, switching to Y_*e*_ partway through time, indicated by the change in color of the vertical lines. Thus, at first X_*e*_'s input fails to evoke spikes in Z_*e*_ since it coincides with the highest inhibition from Z_*i*_ whereas toward the end the spiking activity is driven primarily by X_*e*_, demonstrating the idea behind selective information routing via the CTC mechanism. In row **(E)**, we show the phase differences ϕ_*Xe*_ − ϕ_*Zi*_ = Φ_*ZX*_ and ϕ_*Ye*_ − ϕ_*Zi*_ = Φ_*ZY*_. They gray shaded region indicates the favorable phase difference, which corresponds to aligned peaks between Z_*i*_ and X_*e*_ or Y_*e*_ currents. At the beginning of the displayed data snippet, the network's state is favorable to transfer Y's information, Tr^Y^, switching to be favorable for X, Tr^X^, in the latter portion. **(F)** Distributions of phase differences for different levels of internal noise for the X-Y populations (left) and X-Z (or Y-Z) populations (right). The two peaks of the distributions in the right-hand plot indicate the bistable dynamics of the network. Phase differences between 0 and 0.5π correspond to the preferred state when signal routing should be optimal (shaded in gray). **(G)** The signal content $\bar{SC}$ in the activity of X and Y as a function of internal noise level is displayed. X activity manifested mostly the input signal S_X_ and significantly less of input signal S_Y_, which shows up due to the lateral connection between X and Y. **(H)** The stimulus content of S_X_ input in Z is shown, first without considering the state of the network and then separately for each state, Tr^X^ and Tr^Y^. The errorbars in **(G,H)** correspond to the SE of 10-s simulation runs and the gray shading at the bottom indicates the 95% chance level.

A schematic of the model is presented in Figure 5A. The input (upstream) layer of the model is composed of two oscillators, X and Y, representing two neighboring V1 cortical columns. These are driven by afferent connections delivering independent Poisson spike trains, each modulated by its own input signal, S_X_ and S_Y_. Furthermore, X and Y share a connection from the excitatory pool of neurons of one population to the inhibitory neurons of the other, X_*e*_ to Y_*i*_ and Y_*e*_ to X_*i*_ with connection probability *p*_XY_ = 0.02 and delay τ_XY_ = 5 ms.

In the output (downstream) layer of the model, a third oscillator Z represents a single V4 cortical column that receives input from each of the V1 populations, emulating the convergence of receptive fields when going downstream in the visual system. X_*e*_ and Y_*e*_ project with equal strength onto Z_*e*_ with connection probability *p*_Z_*e*__, and onto Z_*i*_ with connection probability *p*_Z_*i*__. Each of the individual populations retains local parameters of the single cortical column from the previous section, resulting in cyclic activity within the same natural frequency.

Increasing levels of additional background noise significantly increase the firing rates of integrate-and-fire neurons (Brunel and Latham, 2003). Since our model contains all sorts of recurrent connections, these increased firing rates cause various runaway effects that drastically change the behavior of the model. Thus, to make the comparison between different noise levels fair, we scale the driving magnitude of the afferent inputs into each population in order to maintain consistent firing rates across the conditions. First, *S*_*e*_(*t*) and *S*_*i*_(*t*) are scaled for the X and Y oscillators, similar to the case with a single cortical column. Once X and Y evoke the desired output spiking rate of 15 Hz, *p*_Z_*i*__ and *p*_Z_*e*__ are scaled such that Z is sufficiently driven to display the same spiking rates as well.

##### 2.2.1.2. Model dynamics

Due to the intra-population connectivity between X and Y and their associated synaptic delays, their oscillations are consistently in an anti-phase relationship. The outputs from X and Y drive compete to entrain Z, which results in bistable model dynamics as described in Harnack et al. (2015).

To demonstrate these dynamics, we display a snippet of activity in Figures 5B–E, focusing on Z_*e*_. When Z is entrained by X, the troughs of Z_*i*_'s input to Z activity correspond to the peaks of X and to the troughs of Y, and vice versa when Z is entrained by Y (marked by the vertical lines throughout plots Figures 5B–D). Thus, during the first half of the displayed activity, Z_*e*_'s spikes are mostly driven by X's input, and during the second half by Y. These sort of dynamics enact the CTC mechanism to route the input information S_X_ or S_Y_ depending on which population X or Y is in a favorable (matching peaks) phase relationship with Z.

When the peaks of the currents in Z_*e*_ appear aligned, the stable state phase differences φ_*Z*_ − φ_*X*_ = Φ_ZX_ (or φ_*Z*_ − φ_*Y*_ = Φ_ZY_) do not perfectly correspond to 0, but rather span the range between 0 to 0.5π, derived empirically from the model's behavior (shaded in gray in Figure 5E). This is merely an epiphenomenon of the phase extraction, due to the mismatch between the signals' waveforms which stray away from perfect sinusoids.

We designate the system's stable states by using the Φ_ZX_ and Φ_ZY_ phase differences: state Tr^X^ when Z is entrained by X (0 < Φ_ZX_ < 0.5π, corresponding to 25% of available phases) and S_X_ information should be transferred over to Z while S_Y_ is suppressed by Z_*i*_'s inhibition; and state Tr^Y^ (0 < Φ_ZY_ < 0.5π) when the opposite is true. Considering that X and Y are always oscillating in anti-phase, this leaves half of the available phases as the unstable region, when the system is transitioning from one state to the other.

The bistable dynamics of the system are clearly visible in Figure 5F where we plot a histogram of Φ_ZX_ and Φ_ZY_ phase differences, across multiple noise conditions. As can be expected, with increasing levels of noise, the system's affinity to maintain its stable states decreases.

##### 2.2.1.3. Signal transmission

The inherently bistable dynamics provides a perfect mechanism for implementing communication through coherence (CTC). The CTC hypothesis states that when a population receives multiple oscillatory inputs, it can selectively route one and suppress the others by establishing favorable and unfavorable phase relationships, respectively. For example, in the first half of the trial shown in Figures 5B–E, X input to Z_*e*_ arrives when Z_*e*_ is least inhibited by the Z_*i*_ input, putting it into an excitable state and allowing the information content of the signal in X to propagate into (and through) Z. Simultaneously, the bursts of Y's activity arrive concurrently with maximal inhibition from Z_*i*_, hence suppressing Y's information content. In sum, the output spikes of Z during this period primarily reflect the activity it receives from X. The same is true in the other direction—when Y wins the entrainment “battle” over Z, its output propagates onwards, while X's output is effectively suppressed. In the following, we will call these two stable states trans-X-favorable (abbreviated Tr^X^) and trans-Y-favorable (abbreviated Tr^Y^).

By using the spectral coherence, we assess the content of each input signal, S_X_ and S_Y_, in all three populations X, Y, and Z. Due to the recurrent connections between X and Y that were not present in the independent case considered in part I, there is a weak mixing of the input signals in the first layer, as seen in Figure 5G. X represents primarily S_X_ (green bars), and to a small but significant extent S_Y_ (orange bars). For reasons of symmetry, the same lines also represent signal content in Y (green for S_Y_ in Y, and orange for S_X_ in Y).

If we compute the representation of each input signal $\bar{SC}$ in Z output without regard for the current state (Tr^X^ or Tr^Y^), we find that on average each signal is equally expressed, as indicated by the blue bars in Figure 5H. However, when we assess signal content when the network is in the Tr^X^ state, we find that Z activity contains significantly more information from S_X_ (green bars) as opposed to when the system is in Tr^Y^ state (orange bars). Thus, the model does indeed perform signal routing, stochastically switching between the two equivalent input sources. As we increase the background 1/f noise, qualitatively none of these relationships change—populations in a favorable phase relation always route more information. However, at a sufficiently high noise level (σ_*n*_ > 0.15 nA), the difference between stimulus content $\bar{SC}$ during Tr^X^ and Tr^Y^ states is no longer significant (Student's *t*-test between two sets, composed of 100 simulations of 10 s each, *p* > 0.05).

#### 2.2.2. Pulse-Response and State Switch Characteristics of the Columnar Network

As introduced earlier, we will use φ_*i*_ to denote the phase of oscillator *i*, and Δφ_*i*_ to denote the change in phase of oscillator *i* induced by an external pulse. The probability ρ_τ_(Δφ_*i*_|φ_*i*_) to observe a phase shift Δφ_*i*_ a delay τ after a pulse was given when the oscillator was at phase φ_*i*_ then constitutes a stochastic realization of the *phase response-curve* (PRC) of unit *i*.

However, in our extended model an oscillator is part of a network in which a single oscillator's phase is less important for network function than *phase differences* between *pairs* of oscillators. For example, in order to gate an input signal from population X to population Z, their phase difference must be close to 0 as was observed in the previous section. For this reason, we will also consider how the phase difference Φ_*ij*_ = φ_*i*_−φ_*j*_ between populations *i* and *j* is affected by a pulse, giving us a distribution ρ_τ_(ΔΦ_*ij*_|Φ_*ij*_) over induced phase difference shifts ΔΦ_*ij*_. Since these shifts are indicative of changes in the network state, we will use the term “state switch characteristics” for these distributions.

**Figure 6:**
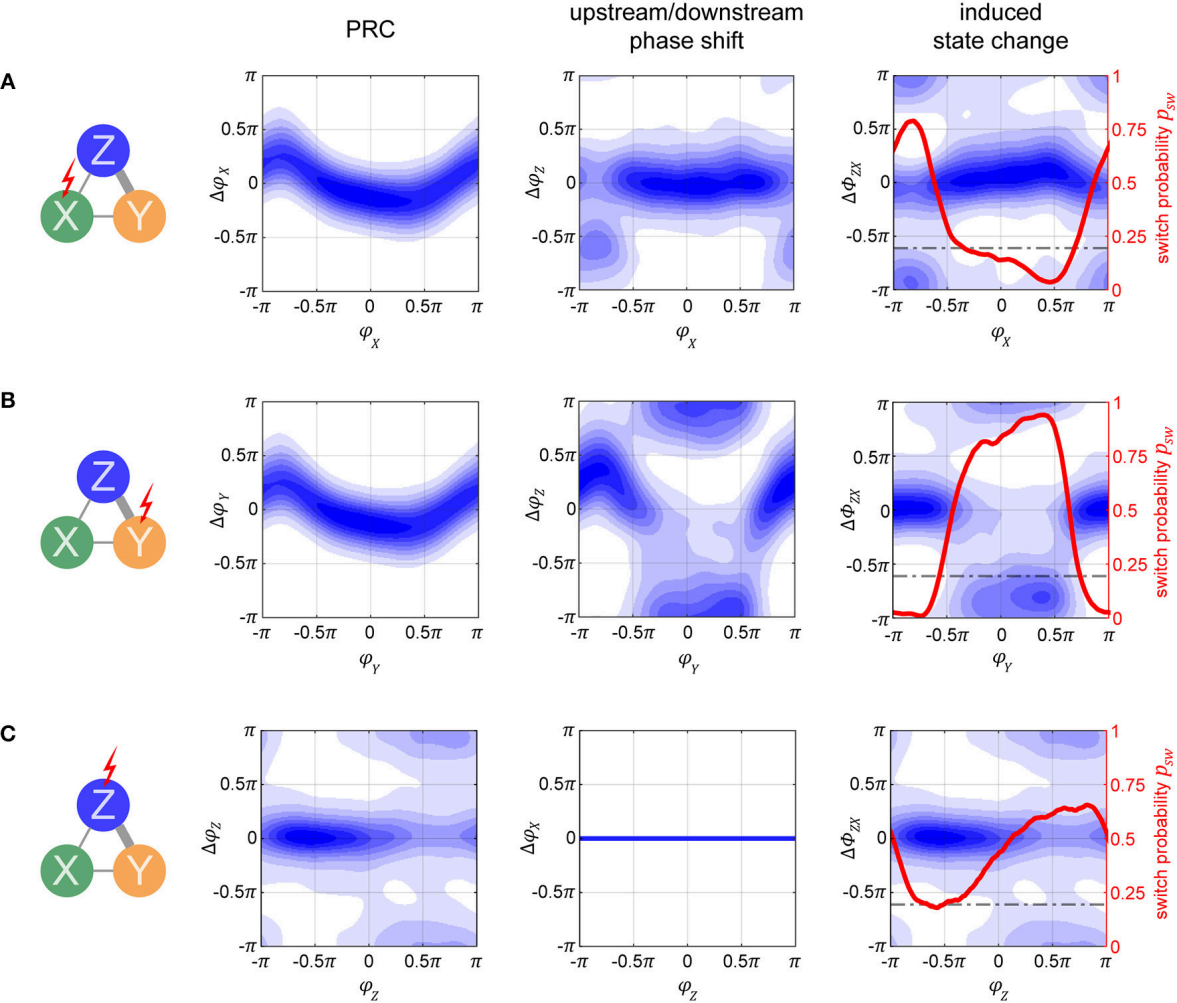
Phase-response and state switch characteristics in the columnar network. Assuming the network being in a Tr^Y^-state, indicated by the thick gray lines in the network diagrams, response characteristics for stimulation of input layer populations X **(A)** and Y **(B)**, as well as for stimulation of output layer population Z **(C)** are displayed. The first column of graphs shows the phase response curves (PRCs) for the stimulated oscillator. Note that these are different from the single oscillator PRCs since the oscillators are now embedded into a larger system. The second column of graphs shows the effect a pulse has on the respective population Z **(A,B)** or population X **(C)**. Blue shading in both columns quantifies the probability density of causing any specific phase shift with darker colors indicating higher probabilities. By taking the difference between the corresponding data points in the densities shown in the left and middle column, we can compute the probability to switch to a Tr^X^ state, which is exemplified in the third column. Since there is no feedback from output population Z to input populations X or Y, the response of X to a pulse onto Z is flat (lower middle graph). The horizontal dashed line in the third column represents the passive switch chance after 100 ms of runtime. Noise level was σ_*n*_ = 0.075 nA for all panels, and pulse strength δ*I* = 1 nA (4 nA) for **(A–C)**.

One can distinguish two conceptually different possibilities to interact with the columnar network: Pulsing population X (or Y) from the input layer, or pulsing population Z in the output layer. In the following paragraphs, we will investigate these two possibilities in more detail, with Figure 6 illustrating the corresponding effects at a delay of τ = 100 ms after the pulse, at an intermediate noise level of σ_*n*_ = 0.075 nA. Furthermore, we assume the network to be in a Tr^Y^ state, and we will thus compute the state switch probabilities to the Tr^X^ state.

##### 2.2.2.1. Pulsing input layer population X or Y

If we apply the pulse to one of the lower level populations, the perturbation will propagate forth and back via recurrent connections and lead to a cascading effect of pulse echos. However, after more time passes (τ = 100 ms is more than sufficient), the X–Y populations settle back into their anti-phase relationship. Because of this, at a sufficient delay τ, the PRC densities for X and Y are essentially identical and appear to resemble a diffused version of the PRC in the single oscillator case (compare the left graphs from Figures 6A,B to the corresponding plot in the middle of Figure 3B).

How do these perturbations act on the output population Z? A pulse given at a peak of X's activity arrives at a trough of Z's activity, giving rise to the phase shifts Δφ_Z_(φ_X_) shown in the middle graph of Figure 6A. However, if a pulse is given at a peak of Y's activity, the propagated pulse arrives at Z at about the same phase as the initial perturbation was given to Y, resulting in the phase shifts Δφ_Z_(φ_Y_) shown in the middle graph in Figure 6B.

How do these different effects of a pulse given to the input layer combine and affect the global state of the columnar network? To obtain the corresponding measure ΔΦ_ZX_, we can take the difference between the corresponding data points from the left and middle graphs in Figures 6A,B, thus obtaining the state switch densities. The corresponding graphs displayed in the right column of Figures 6A,B reveal a bimodal distribution with peaks at 0 and π. In order to best summarize the concentrations in the state switch densities around 0 and π, we calculate the state switch probability *p*_sw_ across the onset phases Φ via $p_{sw}=1-\int_{-\pi /2}^{+\pi /2}\rho (\Delta \Phi )d\Delta \Phi$.

##### 2.2.2.2. Pulsing output layer population Z

Since Z does not send feedback projections to the input layer, the effect of a pulse stays confined exclusively to Z's activity and is independent on the system being in state Tr^X^ or Tr^Y^. Because of this, any phase shift Δφ_Z_ induced onto Z is equivalent to the shifts in phase difference ΔΦ_ZX_ = ΔΦ_ZY_ = Δφ_Z_ between X and Z, and between Z and Y. In Figure 6C we can see that the resulting PRC and state switch distributions are bimodal and have peaks around 0 and π, unlike the effect of a pulse on the single cortical column studied in the previous section. This result is due to the bistable dynamics, which after the immediate effect of the pulse cause Z's phase to continue shifting until one of the stable states is reached. For this reason, this generic behavior is also independent on noise and pulse magnitude. Accordingly, the final phase shift can be either close to zero or close to π, corresponding to no system state change or to a switch between stable states Tr^X^ and Tr^Y^, respectively.

When the pulse magnitude is sufficiently small, e.g., δ*I* ≤ 1 nA, a state change is unlikely (not shown) since the corresponding average phase shift for a single oscillator is too small, Δφ_X_ ≤ 0.5π. Once we increase the pulse strength the likelihood for a phase shift of π increases, with their respective phase onset locations roughly corresponding to the ones which led to Δφ≥0.5π phase shifts in the single oscillator (e.g., compare to Figure 3B, bottom middle plot). Once the strength of the pulse is sufficiently high, a full state reset of the whole system is achieved. Even if the pulse strength is doubled, there are only small changes to the state switch probabilities shown in Figure 6C for δ*I* = 4nA.

Although an initial pulse magnitude of 1 nA was insufficient to obtain a high state switching probability when pulsing Z, when pulsing X with the same strength a much higher switching probability is observed, and for a large range of pulse onset phases (red curves in right column of Figures 6A,B). There are two reasons why switching is easier when targeting an input population: first, the perturbation does not only affect one population but is propagated to all other “players” in the network, and second, for a brief period of time after the pulse, the anti-phase relationship between X and Y is disturbed.

#### 2.2.3. Optimizing Stimulation Pulses for State Switching

Our goal of using stimulation is to cause the network to be continuously in a desired state Tr^X^ or Tr^Y^ for either transmitting signal X or signal Y, respectively. By deriving state switching probabilities from phase-response curves as described in the previous subsection, we now have a tool for optimizing the stimulation pulse parameters toward this goal. Accounting for symmetry between X and Y, all the following results are presented with the aim of switching to a Tr^X^-favorable state. By this design, whenever the network is already in a favorable Tr^X^ relationship, no perturbation is necessary. However, if at any point the network instead is in a Tr^Y^ favorable relationship, we can apply a pulse either to column X, Y, or Z to attempt to switch the state to Tr^X^.

**Figure 7:**
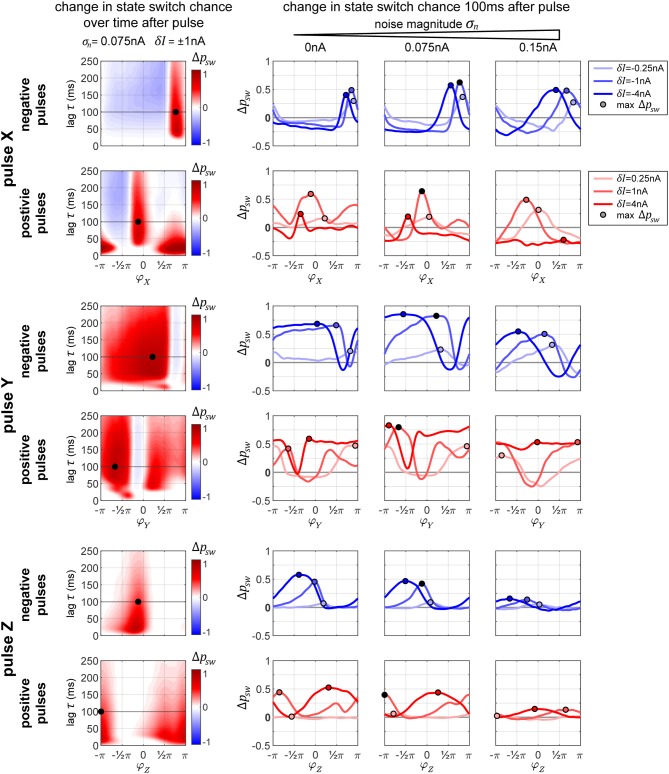
Changes in state switching probability. State switch capabilities for a transition Tr^Y^ → Tr^X^ for the three possible target columns X, Y, and Z of a pulse (upper, middle, and bottom sets of two rows each, respectively) for negative and positive pulses (top and bottom row in each set). The leftmost plot in each row shows how the change in state switch probability Δ*p*_*sw*_ depends on the phase of the pulse onset (horizontal axis) and how it evolves over time (vertical axis going up). The remaining three plots in each row display the switch chance 100 ms after pulse onset, for multiple magnitudes of the pulse (differently colored lines in each graph), and for different background noise levels (separate plot for each noise condition). In each plot, the maximum switch probability is marked by a small circle. The leftmost plot in each row corresponds to one line from the middle plot of the three on the right.

In Figure 7, we display the columnar network's state switch capabilities for Tr^Y^ → Tr^X^ for negative and positive pulses, for each of the three possible pulse-target populations X, Y, and Z. In the plots, we show the *change* in switch probability Δ*p*_*sw*_, since the unpulsed system already possesses a non-zero passive switch probability. In the column on the left, we show how this probability evolves over time (vertical axis) for a medium amount of noise σ_*n*_ = 0.075 nA and medium pulse magnitude δ*I* = ±1 nA in dependence on onset phase φ of the pulse (horizontal axis). In the rightmost three plots in each row, we show the switch probability for multiple pulse magnitudes (different colored lines in each plot) for increasing levels of background noise (the three separate plots). The plots in the column on the left correspond to one line in the middle plot of the three on right. The maximum Δ*p*_*sw*_ is marked in each plot to indicate the optimal onset phase, which can be used in order to switch the system sates. Crucially, in some cases, a pulse leads to a negative Δ*p*_*sw*_ indicating that, if delivered at the wrong moment, a perturbation can actively *hinder* a transition to Tr^X^ and instead stabilize the undesired Tr^Y^-state. In the following paragraphs, we briefly discuss the effects of pulsing the different target columns.

##### 2.2.3.1. Pulse X

The graphs in Figure 7, top two rows, reveal that in addition to having an optimal onset-phase, for each noise condition, there is also an optimal pulse magnitude that results in the largest increase in switching probability, indicated by a small circle. Interestingly, for the medium level of noise, we observe larger switch probabilities than the zero-noise condition.

When applying a negative pulse, there are always intervals of phase onsets that increase, and intervals that decrease the probability of the network switching its state. On the contrary, when applying a positive pulse, at a high noise level (σ_*n*_ = 0.15 nA) and a high pulse magnitude (δ*I* = 4 nA) the onset phase does not appear to matter for the final outcome. In this particular case, all phase onsets lead to a decrease in the switch probability.

The amount of time it takes the network to settle down onto a new state tends to increase with pulse strength (≈30 ms for δ*I* = 0.5 nA pulse vs. ≈60 ms at δ*I* = 2 and 4 nA, not shown in figure). A stronger initial current causes a stronger reverberation of the perturbation, which then takes longer to decay within the system, increasing the time it takes the columnar network to settle back to its normal activity. This effect is particularly strong for the positive pulses, where we get to observe the different phase-states the system goes through before settling down. A negative pulse briefly suppresses all the activity in the network, whereas a positive pulse evokes a volley of spikes in the target population, which then travels and acts as it's own perturbation across the throughout the system.

##### 2.2.3.2. Pulse Y

When pulsing Y instead of X, the graphs in Figure 7, rows 3 and 4, reveal that the switch probabilities appear complementary to the ones from pulsing X. When pulsing X, if a specific phase onset leads to an increase in switching probability, the same phase onset typically leads to a decrease in switching probability if pulsing Y instead. This makes sense, since by changing which population (X or Y) we are pulsing at one specific pulse onset phase, we are essentially changing the onset phase of the propagated pulse that arrives to Z by an amount of π, since X and Y maintain an anti-phase relationship.

Because of this, when applying a positive pulse of a large magnitude (δ*I* = 4 nA) in the noisy condition (σ_*n*_ = 0.15 nA), the probability of a switch is now consistently high across all pulse onsets, whereas in the previous condition a pulse to X was always decreasing switch probability.

##### 2.2.3.3. Pulse Z

As described previously for Figure 6, when pulsing Z, a pulse of low magnitude is hardly sufficient for inducing a significant change in the switch probability. As the noise level of the system increases, the switch probability decreases substantially (Figure 7, bottom two row).

#### 2.2.4. Using Stimulation Pulses to Control Signal Transfer in the Columnar Network

The paradigm to control the synchronization state of the network is similar to controlling the phase of an independent oscillator, with one crucial difference: In the independent oscillator case, a pulse is applied at various phase onsets, depending on what sort of a phase shift is currently necessary. In the columnar network, however, the choice is binary: to switch or not to switch. If we desire to change the current system state, then there is just one specific optimal onset-phase for the pulse. So, for every pulsing condition (i.e., which population pulsed, pulse magnitude, pulse polarity, and network noise level), the state control procedure comes down to the following:

At a time point *t*, apply the stimulation pulse if the following conditions are met

1. Last pulse was more than τ_ref_ ago.
2. The system is in the wrong state and a switch is necessary.
3. The current phase of the pulsed population corresponds to the one that leads to the highest switch probability.

**Figure 8:**
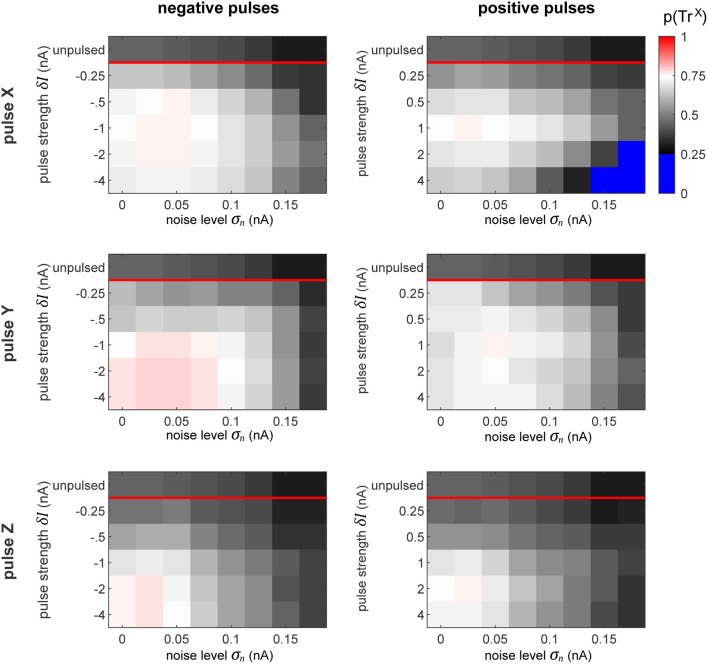
Proportion of time spent in desired state. The plots display the proportion of time that the network spends in the desired state for all the conditions, as labeled. The top row in each subplot indicates the amount of time that the network spends in the desired phase state passively, without any perturbation pulses. Since our favorable state covers a quarter of the full cycle from 0 to 0.5π, the chance level of being in the favorable state is 0.25. To highlight this, the color map turns sharply blue below this value. Thus, the sessions (see Figure 7) whose pulses could only achieve decreasing the switch probability are colored blue.

In order to evaluate how well the pulsing procedure works, we first quantify the proportion of time that the system spends in the desired target state (Figure 8). At the top of each plot, we display the proportion of time that the network spends in that state without pulsing. As defined previously, the desired state is set to the interval Φ_ZX_∈[0, 0.5π], which corresponds to a quarter of the full interval of possible differences.

The goal of the perturbation pulses is to increase this value as much as possible. The results observed in this figure perfectly reflect the corresponding switch chance as predicted by the plots in Figure 7. This is especially clear in the case when we pulse X with using a large positive perturbation (δ*I* ≈ 4 nA) at a high noise level (σ_*n*_ ≈ 0.15 nA). In this condition, the effect of the pulse can only decrease the switch chance, indicated by the blue regions in the top right plot. In all other cases, the procedure succeeds at increasing the amount of time the network spends in the desired state.

Similarly to our previous results from pulsing independent oscillators, the performance of the procedure decreases with increasing noise level. On the contrary, in the columnar network the pulse magnitude and polarity plays a crucial role, whereas in the independent case, the strength of the pulse had no significant effect on the performance. In fact, we observe qualitatively different patterns for which pulse is optimal across the different pulse-polarity and pulsed-target conditions. For instance, when pulsing X, a pulse of δ*I* = −1 or 1 nA achieves the best performances. However, when pulsing Y, negative pulses get better results at higher magnitudes (saturating at sufficiently high levels), whereas positive pulses exhibit lower performance once the magnitudes are sufficiently high.

Generally, with our model's specific setup, the results seem to indicate that pulsing Y (i.e., the population whose information we wish to suppress) provides a much more robust and forgiving conditions, by having more admissible phases of the perturbation onset that result to a state switch.

**Figure 9:**
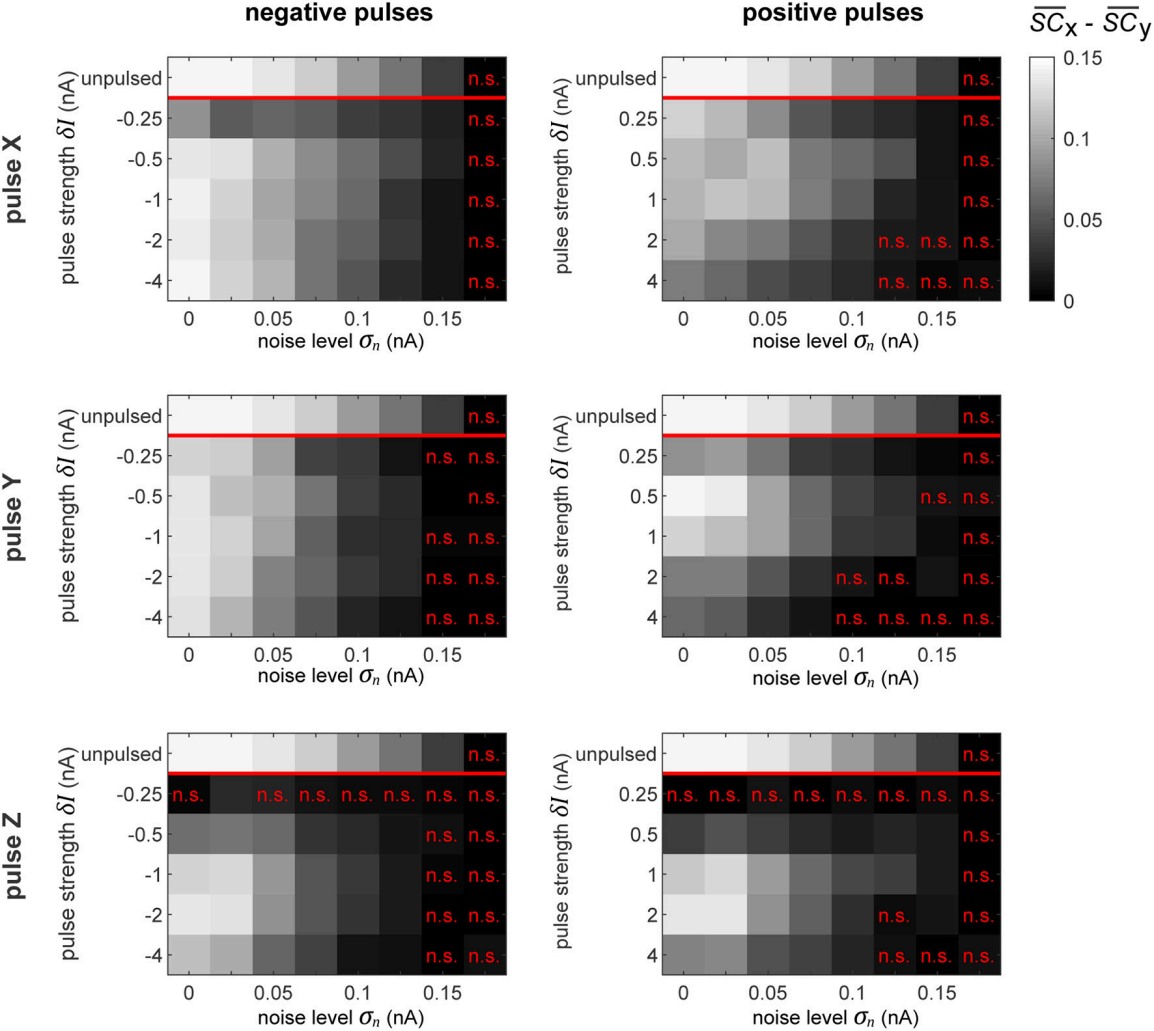
Selective signal routing via precisely pulsing the columnar network. Difference in transferred signal content between the two competing input stimuli S_X_ and S_Y_ for different stimulation paradigms (rows) and pulse polarities (columns), evaluated for different pulse magnitudes δ*I* (vertical axes) and noise levels σ_*n*_ (horizontal axes). The top row in each graph shows the corresponding “optimal” or maximally achievable signal routing performance extracted from time intervals when the network was in a particular preferred state (here, in Tr^X^). Parameter combinations marked by a red “n.s.” indicate conditions where the difference in signal transfer was not significantly different from zero (Student's *T*-test, *p* < 0.05).

Further, we evaluate the signal routing performance of the pulse procedure by evaluating the difference between S_X_ and S_Y_ signal contents in Z. These differences are displayed in Figure 9. Insignificant differences are marked appropriately (*p* > 0.05, Student's *t-*test). At the top of each plot, the maximally achievable difference is displayed, as seen in Figure 5C, by evaluating the signal content in Z without delivering any pulses, but for the time intervals in which the system is spontaneously in the preferred state for transmitting a specific signal.

As we observed in the independent oscillator case, increasing the pulse magnitude increases the amount of degradation of the external signal in the population's activity in all the conditions. In some cases, a pulse of a higher magnitude actually leads to a better performance in terms of keeping the network in the desired state, but simultaneously, it also increases the amount of signal degradation. Since we focus on signal routing differences, the results in Figure 9 reveal the appropriate compromise to achieve the best gating performance.

In a recurrent network, the effect of the pulse on external signal representations is not straightforward and hard to predict. First, the signal represented by the pulsed population is degraded by the injected current. Subsequently, the pulse propagates throughout the rest of the system, causing further degradation of signal representations in *other* populations. Consequently, the signal routing performance crucially depends on which population is pulsed. For instance, if we compare the results in the zero noise condition when pulsing X or Y with a positive pulse of δ*I* = 1 nA, we find that pulsing Y provides much better routing results, even though pulsing X is actually better at establishing the desired network state (see Figure 8). In this case, a significant part of the result is caused by signal S_Y_ getting degraded substantially more than signal S_X_.

## 3. Discussion

### 3.1. Summary

The goal of this study was to investigate how precise perturbations can control a recurrently coupled neural network by using its natural tendency to be in one of several preferred network states. For this purpose we developed a closed-loop paradigm to monitor the system state in realtime and utilized the results to deliver rare, but accurately timed stimulation pulses of proper magnitude. First, we evaluated the method on a structurally simple system—the model of a single cortical column. Here, it was possible to synchronize two independent oscillators up to a critical noise level, and to determine the optimal pulse strength. Next, we applied our paradigm to a more elaborate network of local populations representing recurrently coupled cortical columns, proposed (Harnack et al., 2015) as a prototypical implementation for selective information processing via communication-through-coherence (CTC) in the visual cortex. For successfully interacting with such a system, our results demonstrate that understanding the behavior of one of its constituents in isolation (e.g., by obtaining the phase-response curves, PRCs) is not sufficient—instead one has to probe the network as a whole, which required to compute the probabilities of state switches. Furthermore, we investigated several ways of interacting with the system, targeting either upstream or downstream neural populations. Ultimately, we could simulate the effect of physiological attention and gate signals by bringing the desired population(s) into a preferred (or non-preferred) phase relationship.

### 3.2. Limitation of Model and Significance of Results

Certainly the columnar network is still an abstraction of the real networks performing selective information processing in the visual cortex. We only considered three coupled columns, back-projections from downstream visual areas were not modeled, and we assumed a lateral recurrent coupling structure which is still subject to on-going physiological and anatomical research. Furthermore, we restricted ourselves to investigate ING-oscillators only (for details, see paragraphs below). Additionally, the effects of our perturbation pulses on neural processes are highly simplified in the simulations. However, even when taking these restrictions into account, we believe our work contributes in three important aspects to the field:

- For being successful in interacting with a neural system, the current state of the system *does matter*. This is particularly obvious when trying to construct a visual intracortical prosthesis (Lowery, 2013). Since there is an on-going dynamics in the cortex even in the absence of an actual visual stimulus (Arieli et al., 1996), it is important to know when an artificial stimulus would be most effective, either in inducing a certain percept or in pushing the system into or toward a desired network state. Another requirement is to ensure an ongoing stimulus processing in downstream visual areas. For this purpose, it would be necessary to first bring the network into a state where incoming information can be successfully gated across different stages. This goal was successfully reached in model simulations of our closed-loop stimulation paradigm.
- With respect to selective information processing, we investigate *one specific* of potentially many implementations of the CTC principle. Our results therefore constitute a prediction of how the real network would react if it would work according to our hypothesis. In particular, we predict that pulsing the column representing the unattended stimulus would be very effective in switching between the different network states and in selectively gating a stimulus. This should not be the case if the recurrent interactions would not push the upstream populations X and Y into an antiphase relation, thus providing an opportunity to test this critical assumption.
- Finally, our study brings together the tools needed to establish realtime control of stochastic neural systems. One important insight for us were the severe restrictions imposed by noise, be it intrinsic or on the observation level. Crucially, we present a paradigm that relies on single and rare stimulation pulses, allowing the network to spend the majority of the time unperturbed. This is in opposition to utilizing continuous stimulation or repetitive pulses that explicitly entrain the system, which we argue results in non-natural and forced brain activity. Thus, we conclude that instead of explicitly forcing a network state, it should be of great benefit to account for the system's inherent multistable attractor states and utilize the minimal perturbation to let the network settle naturally in a desired network state.

Below, we discuss the relation of the columnar model to experimental data and possible consequences of changes to the model structure for our stimulation paradigm.

#### 3.2.1. Intra-Population Connectivity

As briefly mentioned in the preceding paragraph, there are two major assumptions in the connectivity between the X,Y and Z modules in the columnar network. First, the connectivity between the lower layer populations X and Y forces them to establish a stable and symmetrical anti-phase relationship between their activities, without establishing a clear winner between the two. Second, there are no back-projections from Z back onto X or Y.

By increasing the connection strength and changing the delays in the X–Y connection it is possible for the populations to synchronize at a different phase, exhibiting the phenomenon of biased competition (Moran and Desimone, 1985) already in the first layer, and controlling the overall bistability of the model. In such a system, the bistable dynamics are evoked as the two populations switch between which one leads and which one follows. The phase response characteristics of such a scenario of two interconnected oscillators have been thoroughly explored in Witt et al. (2013). If we employed this sort of connectivity between X and Y in our model, the winner of the biased competition in the first layer would also entrain Z. As Witt et al. (2013) show, in this scenario one is also able to control the stable state via a precisely timed stimulation pulse.

#### 3.2.2. Local Circuitry

The source of gamma frequency oscillations in the brain has been attributed primarily to two mechanisms: ING—interneuron gamma—which we utilize in our model, and PING—pyramidal interneuron gamma (Tiesinga and Sejnowski, 2009). In the ING mechanism, a population of mutually connected inhibitory neurons generate synchronous IPSPs, creating an ongoing rhythm which is then imposed onto the excitatory neurons (Whittington et al., 1995). In the PING mechanism a volley of excitation stimulates delayed feedback inhibition, resulting in consistent cyclic behavior when the ratio between excitation and inhibition is appropriate. Research shows that both mechanisms can work together to generate gamma frequency oscillations (Brunel and Wang, 2003; Geisler et al., 2005; Belluscio et al., 2012; Buzsáki and Wang, 2012). Either mechanism or a combination of the two constitutes a self-sustaining oscillator and exhibits phase-response characteristics. Hence, we speculate that regardless of the oscillation generating mechanism, the method established in this study can be used to establish desired phase-locking between populations of neurons and route information—however, with a potentially different state switch characteristics.

#### 3.2.3. Transient Synchrony

Even at high noise levels, the rhythmic behavior of our system is an idealized version of what is observed in the visual cortex where the amplitude of oscillations, along with the strength of synchronization phenomena occur as transient events that rarely last longer than 100 ms. In particular, for the V1–V4 interaction explored in this study, gamma activity tends to occur in bursts at theta frequency through phase-amplitude coupling, corresponding to the rate of attentional sampling (Canolty et al., 2006; Landau and Fries, 2012; Spyropoulos et al., 2018). If theta phase amplitude coupling was included in our model, we presume that it should still be possible to control information routing by injecting the appropriate perturbation toward *the beginning* of each theta-coupled gamma burst.

#### 3.2.4. Modeling the Perturbation

In the present study, the applied perturbations involved injecting the same amount of current into all the neurons within a local population. This was designed to model the effect of intracortical microstimulation (ICMS). If we wanted to get closer to the true postsynaptic effect of an ICMS pulse, it would be necessary to work out advanced kernels to convolve with the square wave function that we used. Additionally, it would benefit to have different weights of the perturbations effect by neuron type, physical orientation, and distance to the electrode. As long as the final perturbation is sufficiently short and precise relative to the oscillation cycle, the network dynamics should still exhibit a PRC. We believe that the method employed provides a generic pulse that can be easily modified for other potential stimulation techniques. For example, in the case of modeling an optogenetics pulse (Witt et al., 2013), the stimulation affects just a specific subset of neurons within local population (of type affected by the viral injection of a particular light-sensitive protein).

#### 3.2.5. Robustness: Different Levels of Background Noise Within a Column

The background noise magnitude σ_*n*_ has two effects. First, it masks the flicker signal to be transferred by the excitatory population, and second, it reduces amplitude and frequency of the gamma oscillations generated by the inhibitory population. By allowing for different noise magnitudes $\sigma_{n}^{e}$ and $\sigma_{n}^{i}$, for the excitatory and inhibitory populations, respectively, $\sigma_{n}^{e}$ would therefore predominantly attenuate signal transfer, while $\sigma_{n}^{i}$ would rather reduce gamma amplitude and stability. As long as the noise level remains sufficiently low for the network to still exhibit stable oscillations, the conclusions of the research should not be affected: signal routing by precise perturbations with two different noise levels should work at least equally well, or even better, than in a case where a common noise level of $\sigma_{n}=\text{max}\left(\sigma_{n}^{e},\sigma_{n}^{i}\right)$ is assumed.

#### 3.2.6. Robustness: Different Natural Frequencies Within the Columnar Network

For realizing anti-phase sync between X and Y, and bi-stable synchronization of up- and down-stream populations, a rough match of natural frequencies is important. However, coupled oscillators can tolerate a certain amount of frequency mismatch until (in- or out-of-phase) synchronization breaks down. Stronger couplings allow for larger frequency mismatches, quantified by the width of Arnolds' tongues known from the theory of coupled oscillators (see e.g., Rasband, 2015). For the case of two coupled gamma oscillators realized in PING networks, this property has been thoroughly quantified in Lowet et al. (2015).

Actually, since noise introduces frequency jitter in our model we already have a situation in which the momentary frequencies are always different and can change rapidly, but still bi-stability and entrainment persist until a critical noise level. From these considerations one can conclude that our results are robust against moderate mismatches of the natural frequencies of X, Y, and Z. In particular, the strong coupling from X/Y to Z allows for larger natural frequency deviations between up- and down-stream populations before synchronization breaks down. In any case, asymmetries in natural frequencies induce asymmetries in the preferred states, making one of them more stable than the other.

If natural frequencies are different between populations X and Y, a lopsided leader/follower relationship can emerge, similar to the one reported in Witt et al. (2013). The network as a whole will still be bistable and allow switching between Tr^X^ and Tr^Y^ states, by changing which oscillator leads, and which oscillator follows. Alternatively, X-Y can flip between in-phase and anti-phase states. Here the in-phase state was accompanied with a different working frequency than exhibited by the anti-phase state. We speculate that in this case, perturbation control of signal transmission could still be possible but will be more difficult, since it is essential to prevent in-phase synchronization of X and Y. With even larger natural frequency differences, bistability can vanish completely and making switches between two stable states impossible.

With respect to Z synchronizing to X or Y, in our specific model setup we have observed state switches going “forward,” i.e., Z briefly speeding up, as well as going “backward,” i.e., Z briefly slowing down, in order to switch its entrainment from X to Y or vice versa. In consequence, when X and Y maintain a “proper” anti-phase relationship and Z's natural frequency is different from X and Y, it would favor state switches primarily of the appropriate type: always slowing down if its frequency is sufficiently lower, and speeding up if its oscillation frequency is higher.

When applying our methods to the visual system, it is important to consider two possibilities how differences between natural frequencies could emerge: First, attending one of two competing stimuli can enhance the neural representation of the attended stimulus relative to the representation of the stimulus that has to be ignored. This enhancement can be accompanied by relative rate and/or gamma frequency increases up to 4 Hz (Ray and Maunsell, 2010; Bosman et al., 2012). On this subject, Fries speculates in his 2015 review “Rhythms for Cognition: Communication through Coherence” that after a theta-rhythm evoked phase reset, the faster gamma rhythm would allow the higher frequency V1 population (representing the attended stimulus) have its first burst of activity arrive to V4 prior to the competing V1 population, triggering a wave of inhibition suppressing the inputs from the slower oscillating inputs. Second, differences between two competing stimuli such as their sizes or contrasts could also lead to natural frequency differences. In fact, we expect stimulus manipulations such as a higher contrast of the non-attended stimulus to compete with attentional mechanisms, which might lead to break-down of routing by synchrony if natural frequency mismatches in favor of the non-attended stimulus are becoming too large.

### 3.3. Outlook

Intracortical microstimulation and other methods of providing “artificial” input to the brain (i.e., optogenetics) are a useful tool for investigating neural information processing in a causal manner. More importantly, these techniques can be employed in brain prostheses, helping patients to compensate for disabilities in vision, hearing and touch. In the extreme periphery, devices such as a cochlea or retinal implant have already been successfully deployed. But what about the next stages in the brain? For example, for patients with a damaged optical nerve, an implant must interface primary visual cortex directly. Here, one would have to cope with on-going processes, feedback from higher areas, and a strong recurrent coupling—the state of the system. Overriding these processes and directly providing the stimulus in a “1:1-mapping” is difficult and could exert substantial stress to the tissue, potentially making long-term applications unfeasible (Johnson et al., 1963). We propose that one should rather try to swim with the tide, using the natural tendencies of the network as far as this is possible. In the present study, we started to think about the appropriate strategies and methods and tested them on a very simplistic model, designed after the visual system. The logical next steps could proceed into two directions: first, to put these methods to the test by performing animal experiments, and second, to advance on a theoretical and conceptual level by extending the paradigms into space and time, by delivering complex spatio-temporal stimulation patterns appropriate for a system which exhibits a complex spatio-temporal dynamics even in the absence of an actual stimulus.

## 4. Methods

### 4.1. Neurons and Synapses

Interactions between neurons are governed by synaptic weights ω_*e*_ and ω_*i*_ and conductances *g*_*e*_ and *g*_*i*_, which reflect the magnitude and decay speed of EPSCs (excitatory postsynaptic currents) and IPSCs (negative postsynaptic currents) when receiving a spike from an excitatory or inhibitory cell, respectively.

(4) $$g_{e}(t)=\omega_{e}\sum_{s=1}^{n_{e}}\Theta (t-t_{s,e}-d)\text{exp}\left(\frac{-(t-t_{s,e}-d)}{\tau_{e}}\right)$$

(5) $$\begin{array}{l} g_{i}(t)=\omega_{i}\sum_{s=1}^{n_{i}}\Theta (t-t_{s,i}-d)[\chi_{1}\text{exp}\left(\frac{-(t-t_{s,i}-d)}{\tau_{i}^{1}}\right)\\ \;+\chi_{2}\text{exp}\left(\frac{-(t-t_{s,i}-d)}{\tau_{i}^{2}}\right)] \end{array}$$

Here Θ is the Heaviside function, *d* the synaptic delay, and *t*_*s, e*_ and *t*_*s, i*_ are the times of presynaptic excitatory and inhibitory spikes, respectively. The decay constants for EPSP and IPSP are given by τ_*e*_ and $\tau_{i}^{1,2}$, with the inhibitory response containing a mixture of slow and fast components with the relative contributions controlled by χ_1, 2_. These parameters are set to emulate realistic neurons, in accordance with Bartos et al. (2002) (see Table 1). The activity of the units is simulated in Matlab in discrete time using the forward Euler method with a timestep of *dt* = 0.1*ms*.

### 4.2. Offline Phase Measurement

For measuring the phase of a cyclic signal, we utilize a well-established procedure. For efficiency, the signal is downsampled to 1 kHz and normalized. A power spectrum of the signal is calculated using a Morlet wavelet transform, from which we determine the location and halfway points of the gamma peak. Then, we apply a zero-phase (“filtfilt” command in Matlab) finite-impulse response (FIR) bandpass filter with bandstops at the halfway points found in the power spectrum. This gives us the gamma component of the signal without distorting the phase. Afterwards, the signal is passed through a Hilbert transform (Boashash, 1992), providing us with the complex analytical signal. The argument of the analytical signal gives us the instantaneous gamma phase of the signal. The narrow range of the bandpass filter is necessary, since the instantaneous phase only becomes accurate and meaningful if the filter bandwidth is sufficiently narrow (Nho and Loughlin, 1999).

This sort of a procedure is prone to edge effects and especially to the artifacts induced by sudden spikes in activity due to stimulation pulses used in this study. To decrease the effect of these artifacts, the affected region is set to zero after normalization. Empirical tests showed that the edge and artifact effects on instantaneous phase measurement becomes insignificant around 2 cycles away from the affected region, around 30 ms for gamma oscillations.

### 4.3. Realtime Phase Measurement

When forecasting a discrete time signal *X*_*t*_, given its past time points *X*_*t*−*i*_, an autoregressive model of order *p* is be defined as

$$X_{t}=c+\sum_{i=1}^{p}\alpha_{i}X_{t-i}+\varepsilon_{t}$$

where the set of α_*i*_'s are the parameters of the model, *c* is a constant, and ε_*t*_ is white noise. The parameters and the magnitude of the noise are trained on a pre-existing set of data using the Burg lattice method (“arburg” command in Matlab). The selection of the model order *p* depends on the sampling rate and the characteristics of the input signal and is determined empirically to provide the most accurate phase measurements when compared to the offline phase measurements (McFarland and Wolpaw, 2008).

For speed and efficiency, the AR model was applied to downsampled 1kHz signals, for which the optimal order *p* was found to correspond to the the average number of time steps within a single cycle of oscillatory activity (15 ms for gamma oscillations). For every condition, a separate AR model is trained on an existing 10 s trial, which provides sufficient amount of data to converge on the appropriate AR parameters.

### 4.4. Information Measure Significance Levels

Chance levels for ${\bar{SC}}_{xy}$ are calculated by pairing up the network activity with surrogate input signals. The resulting distribution of SC values allows us to extract the 95th percentile ${\bar{SC}}_{95\%}$, allowing us to evaluate the significance of the information measure score. Further, spectral coherence is affected by sampling size bias. Thus, in order to compare signal routing scores across conditions, they were consistently computed from 100 simulations of 10 s each.

### 4.5. Conditioning of Input Drive on Internal Noise

Increasing the level of noise inherently raises the spiking rates of the affected neurons. Since our network relies on a series of recurrent coupling, a change in mean spiking rate would result in drastically different behavior. Thus, in order to keep the comparison between the different noise levels fair, we scale the magnitude of the mean input drive to the system (Sx and Sy) in order to sustain comparable activity. Thus, in order to obtain comparable model activity between the different internal noise magnitude model conditions, the input drives, *S*_*e*_(*t*) and *S*_*i*_(*t*) were adjusted to achieve a 15Hz average firing rate for the excitatory and 60 Hz for the inhibitory pools of neurons. This was achieved via a simple gradient descent procedure. For example, for a simple oscillator and for the first layer populations X and Y of the bistable model, if the initial drive to the inhibitory neurons led to a firing rate higher (lower) than the desired 60 Hz, the inhibitory drive *S*_*i*_(*t*) was decreased (increased) by an amount proportional to the mean-squared error of the firing rate. Simultaneously, if the excitatory pool's average firing rate was lower (higher) than desired, the excitatory drive *S*_*e*_(*t*) was increased (decreased). The model was then simulated with the updated driving rates and new firing rates were acquired, new firing rate errors were computed and the gradient procedure was repeated until convergence onto the desired firing rate values. In the bistable multi-column model, once the desired firing rates were attained for the X and Y populations, the same procedure was applied to adjust the connection probabilities from X_*e*_ and Y_*e*_ onto Z_*e*_ and Z_*i*_, *p*_Z_*e*__ and *p*_Z_*i*__.

### 4.6. PRC and State Switch Collection Details

To collect the PRC curve data, we simulated 2,500 runs of 1 s duration across all the possible conditions (noise level, pulse strength, which population pulsed). In each run, the perturbation occured at 0.5 s, providing us with enough signal before and after the pulse to extract the relevant phase information. In addition, we added a control group where the pulse magnitude is set to 0—no pulse. For each run, we computed the offline phase across the entire trial. In addition, we determined the instantaneous phase at stimulation onset φ by using the AR signal prediction procedure to avoid any artifacts caused by the pulse. By pairing up the appropriate trials between the pulsed and the control groups, we calculated the phase difference ΔΦ between the unpulsed and pulsed runs at time τ after *t*_*onset*_. In the independent oscillator case, the pairing process was only concerned with putting trials together with a minimal difference between their corresponding values of φ at pulse onset. In the bistable columnar network, the AR procedure was used to evaluate both the onset phase of the pulsed population as well as the phase-state difference Z-X and Z-Y at pulse onset, with the pairing procedure accounting for both, the network state and the stimulation pulse onset phase.

## Author Contributions

DL contributed to the concept of the work, modeling, and analysis of data, and draft of the manuscript. UE contributed to the concept of the work, supervision, and draft and revision of the manuscript.

### Conflict of Interest Statement

The authors declare that the research was conducted in the absence of any commercial or financial relationships that could be construed as a potential conflict of interest.

## Footnotes

^1^In our mathematical notation, we consistently designate *phases* with φ, *phases shifts* (induced by the perturbation) with a prefix Δ, and *phase differences* (between oscillators *i* and *j*) with a greek uppercase Φ_*ij*_. Consequently, ΔΦ_XY_ will describe the phase shift induced by a pulse on the phase difference between oscillators X and Y.

## References

[B1] Abbott L. Kepler T. B. (1990). Model neurons: from Hodgkin-Huxley to Hopfield, in Statistical Mechanics of Neural Networks, ed Garrido L (Berlin; Heidelberg: pringer), 5–18.

[B2] Akam T. Oren I. Mantoan L. Ferenczi E. Kullmann D. M. (2012). Oscillatory dynamics in the hippocampus support dentate gyrus–CA3 coupling. Nat. Neurosci. 15, 763–768. 10.1038/nn.3081 22466505 PMC3378654

[B3] Arieli A. Sterkin A. Grinvald A. Aertsen A. (1996). Dynamics of ongoing activity: explanation of the large variability in evoked cortical responses. Science 273, 1868–1871. 10.1126/science.273.5283.1868 8791593

[B4] Bartos M. Vida I. Frotscher M. Meyer A. Monyer H. Geiger J. R.. (2002). Fast synaptic inhibition promotes synchronized gamma oscillations in hippocampal interneuron networks. Proc. Natl. Acad. Sci. U.S.A. 99, 13222–13227. 10.1073/pnas.192233099 12235359 PMC130614

[B5] Belluscio M. A. Mizuseki K. Schmidt R. Kempter R. Buzsáki G. (2012). Cross-frequency phase–phase coupling between theta and gamma oscillations in the hippocampus. J. Neurosci. 32, 423–435. 10.1523/JNEUROSCI.4122-11.2012 22238079 PMC3293373

[B6] Benabid A. L. Chabardes S. Mitrofanis J. Pollak P. (2009). Deep brain stimulation of the subthalamic nucleus for the treatment of Parkinson's disease. Lancet Neurol. 8, 67–81. 10.1016/S1474-4422(08)70291-6 19081516

[B7] Berényi A. Belluscio M. Mao D. Buzsáki G. (2012). Closed-loop control of epilepsy by transcranial electrical stimulation. Science 337, 735–737. 10.1126/science.1223154 22879515 PMC4908579

[B8] Blinowska K. J. Malinowski M. (1991). Non-linear and linear forecasting of the eeg time series. Biol. Cybern. 66, 159–165. 10.1007/BF00243291 1768720

[B9] Boashash B. (1992). Estimating and interpreting the instantaneous frequency of a signal. II. Algorithms and applications. Proc. IEEE 80, 540–568. 10.1109/5.135378

[B10] Bosman C. A. Schoffelen J.-M. Brunet N. Oostenveld R. Bastos A. M. Womelsdorf T.. (2012). Attentional stimulus selection through selective synchronization between monkey visual areas. Neuron 75, 875–888. 10.1016/j.neuron.2012.06.037 22958827 PMC3457649

[B11] Brunel N. Latham P. E. (2003). Firing rate of the noisy quadratic integrate-and-fire neuron. Neural Comput. 15, 2281–2306. 10.1162/089976603322362365 14511522

[B12] Brunel N. Wang X.-J. (2003). What determines the frequency of fast network oscillations with irregular neural discharges? I. Synaptic dynamics and excitation-inhibition balance. J. Neurophysiol. 90, 415–430. 10.1152/jn.01095.2002 12611969

[B13] Buzsáki G. Wang X.-J. (2012). Mechanisms of gamma oscillations. Annu. Rev. Neurosci. 35, 203–225. 10.1146/annurev-neuro-062111-150444 22443509 PMC4049541

[B14] Canavier C. C. (2015). Phase-resetting as a tool of information transmission. Curr. Opin. Neurobiol. 31, 206–213. 10.1016/j.conb.2014.12.003 25529003 PMC4375052

[B15] Canolty R. T. Edwards E. Dalal S. S. Soltani M. Nagarajan S. S. Kirsch H. E.. (2006). High gamma power is phase-locked to theta oscillations in human neocortex. Science 313, 1626–1628. 10.1126/science.1128115 16973878 PMC2628289

[B16] Cardin J. A. Carlén M. Meletis K. Knoblich U. Zhang F. Deisseroth K.. (2009). Driving fast-spiking cells induces gamma rhythm and controls sensory responses. Nature 459, 663–667. 10.1038/nature08002 19396156 PMC3655711

[B17] Chen L. L. Madhavan R. Rapoport B. I. Anderson W. S. (2013). Real-time brain oscillation detection and phase-locked stimulation using autoregressive spectral estimation and time-series forward prediction. IEEE Trans. Biomed. Eng. 60, 753–762. 10.1109/TBME.2011.2109715 21292589 PMC3371105

[B18] Coenen A. Fine E. Zayachkivska O. (2014). Adolf Beck: a forgotten pioneer in electroencephalography. J. Hist. Neurosci. 23, 276–286. 10.1080/0964704X.2013.867600 24735457

[B19] Fenno L. Yizhar O. Deisseroth K. (2011). The development and application of optogenetics. Annu. Rev. Neurosci. 34, 389–412. 10.1146/annurev-neuro-061010-113817 21692661 PMC6699620

[B20] Fisher R. Salanova V. Witt T. Worth R. Henry T. Gross R.. (2010). Electrical stimulation of the anterior nucleus of thalamus for treatment of refractory epilepsy. Epilepsia 51, 899–908. 10.1111/j.1528-1167.2010.02536.x 20331461

[B21] Fourcaud N. Brunel N. (2002). Dynamics of the firing probability of noisy integrate-and-fire neurons. Neural Comput. 14, 2057–2110. 10.1162/089976602320264015 12184844

[B22] Fries P. (2005). A mechanism for cognitive dynamics: neuronal communication through neuronal coherence. Trends Cogn. Sci. 9, 474–480. 10.1016/j.tics.2005.08.011 16150631

[B23] Fries P. (2015). Rhythms for cognition: communication through coherence. Neuron 88, 220–235. 10.1016/j.neuron.2015.09.034 26447583 PMC4605134

[B24] Geisler C. Brunel N. Wang X.-J. (2005). Contributions of intrinsic membrane dynamics to fast network oscillations with irregular neuronal discharges. J. Neurophysiol. 94, 4344–4361. 10.1152/jn.00510.2004 16093332

[B25] Grothe I. Neitzel S. D. Mandon S. Kreiter A. K. (2012). Switching neuronal inputs by differential modulations of gamma-band phase-coherence. J. Neurosci. 32, 16172–16180. 10.1523/JNEUROSCI.0890-12.2012 23152601 PMC6794021

[B26] Grothe I. Rotermund D. Neitzel S. D. Mandon S. Ernst U. A. Kreiter A. K.. (2018). Attention selectively gates afferent signal transmission to area V4. J. Neurosci. 38, 3441–3452. 10.1523/JNEUROSCI.2221-17.2018 29618546 PMC6596051

[B27] Guevara Erra R. Perez Velazquez J. L. Rosenblum M. (2017). Neural synchronization from the perspective of non-linear dynamics. Front. Comput. Neurosci. 11:98. 10.3389/fncom.2017.00098 29123478 PMC5662639

[B28] Harnack D. Ernst U. A. Pawelzik K. R. (2015). A model for attentional information routing through coherence predicts biased competition and multistable perception. J. Neurophysiol. 114, 1593–1605. 10.1152/jn.01038.2014 26108958 PMC4563023

[B29] Johnson A. Klassen G. McGregor M. Dobell A. (1963). Long-term electrical stimulation of the heart in Stokes-Adams disease. Can. Med. Assoc. J. 89, 683–686. 14055828 PMC1922003

[B30] Landau A. N. Fries P. (2012). Attention samples stimuli rhythmically. Curr. Biol. 22, 1000–1004. 10.1016/j.cub.2012.03.054 22633805

[B31] Logothetis N. K. Augath M. Murayama Y. Rauch A. Sultan F. Goense J.. (2010). The effects of electrical microstimulation on cortical signal propagation. Nat. Neurosci. 13, 1283–1291. 10.1038/nn.2631 20818384

[B32] Lowery A. J. (2013). Introducing the monash vision group's cortical prosthesis, in 2013 20th IEEE International Conference on Image Processing (ICIP) (Piscataway, NJ), 1536–1539.

[B33] Lowet E. Roberts M. Hadjipapas A. Peter A. van der Eerden J. De Weerd P. (2015). Input-dependent frequency modulation of cortical gamma oscillations shapes spatial synchronization and enables phase coding. PLoS Comput. Biol. 11:e1004072. 10.1371/journal.pcbi.1004072 25679780 PMC4334551

[B34] Martin J. L. R. Barbanoj M. J. Schlaepfer T. E. Thompson E. Pérez V. Kulisevsky J. (2003). Repetitive transcranial magnetic stimulation for the treatment of depression: systematic review and meta-analysis. Br. J. Psychiatry 182, 480–491. 10.1192/bjp.182.6.480 12777338

[B35] McFarland D. J. Wolpaw J. R. (2008). Sensorimotor rhythm-based brain–computer interface (BCI): model order selection for autoregressive spectral analysis. J. Neural Eng. 5, 155–162. 10.1088/1741-2560/5/2/006 18430974 PMC2747265

[B36] Moran J. Desimone R. (1985). Selective attention gates visual processing in extrastriate cortex. Science 229, 782–784. 4023713 10.1126/science.4023713

[B37] Mortezapouraghdam Z. Corona-Strauss F. I. Takahashi K. Strauss D. J. (2018). Reducing the effect of spurious phase variations in neural oscillatory signals. Front. Comput. Neurosci. 12:82. 10.3389/fncom.2018.00082 30349470 PMC6186847

[B38] Nho W. Loughlin P. J. (1999). When is instantaneous frequency the average frequency at each time? IEEE Signal Process. Lett. 6, 78–80. 10.1109/97.752059

[B39] Ni J. Wunderle T. Lewis C. M. Desimone R. Diester I. Fries P. (2016). Gamma-rhythmic gain modulation. Neuron 92, 240–251. 10.1016/j.neuron.2016.09.003 27667008 PMC5053905

[B40] Packer A. M. Yuste R. (2011). Dense, unspecific connectivity of neocortical parvalbumin-positive interneurons: a canonical microcircuit for inhibition? J. Neurosci. 31, 13260–13271. 10.1523/JNEUROSCI.3131-11.2011 21917809 PMC3178964

[B41] Prinz A. A. Thirumalai V. Marder E. (2003). The functional consequences of changes in the strength and duration of synaptic inputs to oscillatory neurons. J. Neurosci. 23, 943–954. 10.1523/JNEUROSCI.23-03-00943.2003 12574423 PMC6741924

[B42] Rasband S. N. (2015). Chaotic Dynamics of Nonlinear Systems. Mineola, NY: Courier Dover Publications.

[B43] Ray S. Maunsell J. H. (2010). Differences in gamma frequencies across visual cortex restrict their possible use in computation. Neuron 67, 885–896. 10.1016/j.neuron.2010.08.004 20826318 PMC3001273

[B44] Sarnthein J. Petsche H. Rappelsberger P. Shaw G. Von Stein A. (1998). Synchronization between prefrontal and posterior association cortex during human working memory. Proc. Natl. Acad. Sci. U.S.A. 95, 7092–7096. 10.1073/pnas.95.12.7092 9618544 PMC22750

[B45] Schmidt K. E. Goebel R. Löwel S. Singer W. (1997). The perceptual grouping criterion of colinearity is reflected by anisotropies of connections in the primary visual cortex. Eur. J. Neurosci. 9, 1083–1089. 10.1111/j.1460-9568.1997.tb01459.x 9182961

[B46] Schultheiss N. W. Prinz A. A. Butera R. J. (2011). Phase Response Curves in Neuroscience: Theory, Experiment, and Analysis. New York, NY: Springer Science & Business Media.

[B47] Siegle J. H. Pritchett D. L. Moore C. I. (2014). Gamma-range synchronization of fast-spiking interneurons can enhance detection of tactile stimuli. Nat. Neurosci. 17, 1371–1379. 10.1038/nn.3797 25151266 PMC4229565

[B48] Singer W. (1999). Neuronal synchrony: a versatile code for the definition of relations? Neuron 24, 49–65. 10.1016/S0896-6273(00)80821-1 10677026

[B49] Smeal R. M. Ermentrout G. B. White J. A. (2010). Phase-response curves and synchronized neural networks. Philos. Trans. R. Soc. Lond. B Biol. Sci. 365, 2407–2422. 10.1098/rstb.2009.0292 20603361 PMC2894948

[B50] Spyropoulos G. Bosman C. A. Fries P. (2018). A theta rhythm in macaque visual cortex and its attentional modulation. Proc. Natl. Acad. Sci. U.S.A. 115, E5614–E5623. 10.1073/pnas.1719433115 29848632 PMC6004461

[B51] Stepanyants A. Martinez L. M. Ferecskó A. S. Kisvárday Z. F. (2009). The fractions of short-and long-range connections in the visual cortex. Proc. Natl. Acad. Sci. U.S.A. 106, 3555–3560. 10.1073/pnas.0810390106 19221032 PMC2651285

[B52] Stettler D. D. Das A. Bennett J. Gilbert C. D. (2002). Lateral connectivity and contextual interactions in macaque primary visual cortex. Neuron 36, 739–750. 10.1016/S0896-6273(02)01029-2 12441061

[B53] Tehovnik E. Tolias A. Sultan F. Slocum W. Logothetis N. (2006). Direct and indirect activation of cortical neurons by electrical microstimulation. J. Neurophysiol. 96, 512–521. 10.1152/jn.00126.2006 16835359

[B54] Tiesinga P. Sejnowski T. J. (2009). Cortical enlightenment: are attentional gamma oscillations driven by ING or PING? Neuron 63, 727–732. 10.1016/j.neuron.2009.09.009 19778503 PMC2778762

[B55] Tsodyks M. Kenet T. Grinvald A. Arieli A. (1999). Linking spontaneous activity of single cortical neurons and the underlying functional architecture. Science 286, 1943–1946. 10.1126/science.286.5446.1943 10583955

[B56] Uhlhaas P. J. Singer W. (2006). Neural synchrony in brain disorders: relevance for cognitive dysfunctions and pathophysiology. Neuron 52, 155–168. 10.1016/j.neuron.2006.09.020 17015233

[B57] Varela F. Lachaux J.-P. Rodriguez E. Martinerie J. (2001). The brainweb: phase synchronization and large-scale integration. Nat. Rev. Neurosci. 2, 229–239. 10.1038/35067550 11283746

[B58] Velazquez J. P. Erra R. G. Rosenblum M. (2015). The epileptic thalamocortical network is a macroscopic self-sustained oscillator: evidence from frequency-locking experiments in rat brains. Sci. Rep. 5:8423. 10.1038/srep08423 25672543 PMC4325330

[B59] Velazquez J. P. Galan R. Dominguez L. G. Leshchenko Y. Lo S. Belkas (2007). Phase response curves in the characterization of epileptiform activity. Phys. Rev. E 76:061912 10.1103/PhysRevE.76.061912 18233874

[B60] Vinck M. Womelsdorf T. Buffalo E. A. Desimone R. Fries P. (2013). Attentional modulation of cell-class-specific gamma-band synchronization in awake monkey area V4. Neuron 80, 1077–1089. 10.1016/j.neuron.2013.08.019 24267656 PMC3840396

[B61] Voloh B. Womelsdorf T. (2016). A role of phase-resetting in coordinating large scale neural networks during attention and goal-directed behavior. Front. Syst. Neurosci. 10:18. 10.3389/fnsys.2016.00018 27013986 PMC4782140

[B62] Whittington M. A. Traub R. D. Jefferys J. G. (1995). Synchronized oscillations in interneuron networks driven by metabotropic glutamate receptor activation. Nature 373:612. 10.1038/373612a0 7854418

[B63] Witt A. Palmigiano A. Neef A. El Hady A. Wolf F. Battaglia D. (2013). Controlling the oscillation phase through precisely timed closed-loop optogenetic stimulation: a computational study. Front. Neural Circuits 7:49. 10.3389/fncir.2013.00049 23616748 PMC3627980

[B64] Wurtz R. H. (2015). Using perturbations to identify the brain circuits underlying active vision. Philos. Trans. R. Soc. B 370:20140205. 10.1098/rstb.2014.0205 26240420 PMC4528817

